# Cytokine-induced PD-L1 and PD-L2 expression is preserved but reverse signalling is altered in rheumatoid arthritis fibroblast-like synoviocytes

**DOI:** 10.1186/s13075-026-03841-7

**Published:** 2026-06-02

**Authors:** Tilia Selldén, Anna-Karin Hultgård Ekwall, Georgios Chatziagorou, Anna-Carin Lundell, Anna Rudin

**Affiliations:** 1 Department of Rheumatology and Inflammation Research, Institute of Medicine, Sahlgrenska Academy, University of Gothenburg, Box 480, S-405 30 Gothenburg, Sweden; 2 Clinical Rheumatology Research Center, Sahlgrenska University Hospital, Gothenburg, Sweden; 3 SportsMed Orthopaedic Clinic, Carlanderska Hospital, Gothenburg, Sweden; 4 Department of Orthopedics, Institute of Clinical Sciences, Sahlgrenska Academy, University of Gothenburg, Gothenburg, Sweden

**Keywords:** PD-L1, PD-L2, Reverse signalling, Fibroblast-like synoviocytes, Rheumatoid arthritis

## Abstract

**Background:**

Dysfunctional programmed cell death-1 (PD1) signalling may contribute to persistent immune activation in rheumatoid arthritis (RA). However, co-inhibitory molecule interactions between T cells and fibroblast-like synoviocytes (FLS) remain insufficiently understood. This study aimed to determine whether T cell activation and subset-associated cytokines differentially regulate the expression of PD-ligand 1 (PD-L1) and PD-L2 on FLS from RA patients compared with non-inflammatory (NI) controls, and whether PD1 engagement induces distinct transcriptional downstream responses in RA-FLS versus NI-FLS.

**Methods:**

Primary FLS cell lines were cultured from synovial tissue of patients with established RA or NI controls undergoing arthroscopy due to previous injury. Cells were cultured with CD3+ T cells or stimulated with TNF, IFNγ, IL-4, or TGF-β, and PD-L1 and PD-L2 expression was analysed by flow cytometry. PD-L1 and PD-L2 knockout FLS were generated using CRISPR/Cas9. TNF-primed knockout or mock-transfected RA- and NI-FLS were treated with soluble PD1, and transcriptomic responses were analysed by total mRNA sequencing.

**Results:**

Analysis of publicly available RNA-sequencing datasets showed that, in early RA, PD-L1 and PD-L2 expression were significantly higher in lymphoid compared to myeloid and fibroid synovial tissue pathotypes. In established RA, PD-L1 and PD-L2 expression in FLS showed a trend towards higher levels in lymphocyte-rich compared to lymphocyte-poor tissue. In vitro, activated but not resting T cells upregulated both PD-L1 and PD-L2 on FLS. Further, TNF and IFNγ induced PD-L1 expression up to fourfold compared with unstimulated FLS, whereas IL-4 and TGF-β had no effect. PD-L2 was induced by TNF, IFNγ, and IL-4 to similar levels. PD-L1 and PD-L2 expression did not differ between RA- and NI-FLS either in co-culture with T cells or under cytokine stimulation. Finally, soluble PD1-induced reverse signalling in FLS altered the expression of threefold more genes in NI-FLS compared to RA-FLS, indicating that RA-FLS might be less responsive to PD1 signalling. PD-L1 or PD-L2 knockout further showed that reverse signalling through each ligand modulates distinct gene sets in FLS.

**Conclusions:**

Cytokine-induced PD-L1 and PD-L2 expression is preserved in FLS from established RA but PD1-ligand signalling is altered, potentially contributing to the loss of immune regulation in RA.

**Supplementary Information:**

The online version contains supplementary material available at 10.1186/s13075-026-03841-7.

## Introduction

Rheumatoid arthritis (RA) is a chronic systemic autoimmune disease characterized by progressive joint inflammation that leads to pain, swelling, and irreversible damage if untreated. However, RA is a clinically and immunologically heterogeneous disorder, and patterns of immune cell infiltration within the joints vary among patients. In early RA (eRA), about half of patients display organized B and T cell infiltrates that form ectopic lymphoid-like structures (lymphoid pathotype), whereas others show presence of macrophages and monocytes (myeloid pathotype) or minimal immune cell infiltration (pauci-immune/fibroid pathotype) [1]. Within the RA synovium, fibroblast-like synoviocytes (FLS) become activated by infiltrating immune cells and, in turn, amplify local inflammation by producing pro-inflammatory mediators [2] and through direct interactions with T cells [3, 4]. These reciprocal interactions initiate and sustain chronic inflammation within the joint. While T cell communication with antigen-presenting cells (APCs) is well characterized, the mechanisms regulating T cell–FLS crosstalk, particularly those involving co-inhibitory signalling pathways, remain insufficiently understood.

Programmed death-ligand 1 (PD-L1) and PD-L2 are two co-inhibitory ligands that suppress T cell proliferation and activation when they bind to their receptor programmed cell death 1 (PD1). In healthy individuals, PD-L1 is broadly expressed on immune cells, including granulocytes, macrophages, T cells and B cells, whereas PD-L2 is primarily found on granulocytes and macrophages. Both ligands are also constitutively present in peripheral tissues on non-immune cells, including endothelial cells and fibroblasts (Human Protein Atlas proteinatlas.org, [5]). During tissue injury and inflammation, PD-L1 and PD-L2 levels become elevated on both immune and non-immune cells [6–8]. At the transcriptional level, PD-L1 and PD-L2 expression is upregulated in synovial tissue from patients with arthralgia, undifferentiated arthritis, eRA, and established RA compared with osteoarthritis and healthy controls (HC) [9]. However, it remains to be clarified whether FLS express these ligands in vivo. In vitro, surface expression of PD-L1 and PD-L2 increases on RA-FLS and on monocytes and dendritic cells from healthy donors in response to tumour necrosis factor (TNF) and interferon gamma (IFNγ) stimulation [6–8]. TNF and IFNγ are, however, not present in the synovial fluid in individuals with early inflammatory arthritis who subsequently develop RA; instead, cytokines related to T-helper (Th) 2 and Th17 responses, including IL-4, IL-13, and IL-17, dominate the synovial fluid of these patients [10]. We have previously shown that Th1, Th2, and Th17-related cytokines induce the production of distinct pro-inflammatory cytokines and chemokines in RA-FLS [2]. However, whether Th-related cytokines regulate PD-L1 and/or PD-L2 expression on FLS from RA patients and whether expression levels differ from non-inflammatory (NI) controls remain unknown, as no study has compared cytokine-induced ligand expression between RA-FLS and NI-FLS.

Although the effects on T cells mediated by the interaction of PD-L1 and PD-L2 with PD1 have been extensively studied in RA [11–13], the reverse effects of this interaction, i.e., signalling downstream of PD-L1 and PD-L2, have received limited attention. In cancer, PD-L1 reverse signalling enhances resistance to IFNγ, cell survival and metabolic activity of melanoma and Hodgkin lymphoma cells, thereby promoting immune evasion in addition to suppressing T cell activation via PD1-mediated co-inhibition [14, 15]. Both membrane-bound and soluble PD1 (sPD1) were capable of triggering PD-L1 reverse signalling in tumour cells [15]. In autoimmunity, no studies in mice or humans have investigated whether reverse PD-L1 or PD-L2 signalling occurs in disease-related cells, or whether these ligands activate distinct downstream transcriptional pathways upon PD1 engagement.

PD1 is expressed on the majority of infiltrating CD4+ and CD8+ T cells within the synovial tissue in both early and established RA [9, 16] and is essential for suppressing the activation of autoreactive T cells in autoimmune disease [17]. However, the persistence of synovial inflammation and progression from early to established RA suggests that PD1-mediated immune regulation is impaired. In this study, we first aimed to assess whether the PD1 ligands are accessible on FLS to T cells during inflammatory conditions by evaluating PD-L1 and PD-L2 surface expression on RA-FLS compared with NI-FLS when exposed to T cells or T cell-associated cytokines. Secondly, we aimed to investigate whether engagement of PD1 with PD-L1 and PD-L2 induces distinct global transcriptional patterns in RA-FLS relative to NI-FLS, to address whether disease-related alterations in FLS affect downstream reverse signalling through these ligands.

## Methods

### Publicly available datasets and patient cohorts

In this study, data from three publicly available RNA sequencing datasets were utilized: (1) a bulk RNA-seq dataset from the Pathobiology of Early Arthritis Cohort (PEAC) study, containing synovial tissue samples from treatment-naïve eRA patients with a symptom duration of less than 12 months (data from https://peac.hpc.qmul.ac.uk; original data files are found on ArrayExpress #E-MTAB-6141) [1], (2) a single-cell RNA-seq dataset from the Accelerating Medicines Partnership Rheumatoid Arthritis (AMP-RA) program, comprising synovial tissue samples from patients with established RA (ImmPort: SDY998) [18]; and (3) a bulk RNA-seq dataset including synovial tissue samples from both treatment-naïve eRA and established RA patients (GEO database: GSE89408) [9]. Detailed descriptions of sample collection are found in the original publications. Patient samples used in the present study for experimental investigations at our laboratory were collected as part of the SYNBIO study (synovial biopsies from NI controls and established RA) and the SYNFLUID study (blood and synovial fluid from established RA), as further described in detail below.

### Synovial tissue samples

Synovial tissue samples were obtained from RA patients (*n* = 7) diagnosed according to either the American College of Rheumatology (ACR) 1987 or European Alliance of Associations for Rheumatology (EULAR) 2010 classification criteria undergoing arthroplasty or synovial biopsy at Sahlgrenska University Hospital, Gothenburg, Sweden. Patients had to have clinically active RA, be ≥ 18 years of age, and have stopped immunosuppressive treatment, including methotrexate, two weeks before surgery. Synovial tissue samples were also obtained from NI controls (*n* = 7) undergoing arthroscopy, due to previous injury to the cruciate ligament or to the meniscus, at Carlanderska Hospital, Gothenburg, Sweden. Key inclusion criteria were ≥ 18 years of age and no sign of arthritis at the time of inclusion. Patient characteristics are shown in Supplementary Tables 1 and 2. The procedures were approved by the Ethics Committee of Gothenburg (SYNBIO study: Dnr 1087–16 with amendment 2023–01470-02), and all patients gave written informed consent.

### Isolation and expansion of fibroblast-like synoviocytes

Synovial tissue samples were dissected into 1–2 mm segments and transferred to a tube containing 5 ml of Dulbecco’s modified Eagle’s medium (DMEM) GlutaMAX™ (Life Technologies Inc., Carlsbad, CA, USA) with 25 μg/ml of Liberase™ TM (Roche, Mannheim, Germany) and incubated for 60 min. Dissolved tissue was rinsed twice in phosphate-buffered saline (PBS; HyClone™, GE Healthcare, Chicago, USA) and transferred into a culture flask containing 5 ml of complete media, i.e. DMEM GlutaMAX™ supplemented with 10% heat-inactivated fetal bovine serum (FBS; Life Technologies Inc.), 0.5% Penicillin–Streptomycin (Life Technologies Inc.; 10,000 U/mL stock concentration), and 0.1% gentamicin (Sigma-Aldrich, St. Louis, MO, USA; 50 mg/mL stock concentration). FLS were incubated at 37 °C at 5% CO2 and cultured until passage 3 before use. Cells in passages 3–6 were used in experiments. Cell lines used in the same experiments were similar in passage number.

### Quantification of soluble PD1 in blood and synovial fluid

Paired blood and synovial fluid samples were collected from patients with established RA (*n* = 12) who were diagnosed according to the ACR/EULAR 2010 classification criteria at the Rheumatology Clinic, Sahlgrenska University Hospital, Gothenburg, Sweden. Key inclusion criteria were ≥ 18 years of age and active disease with ≥ one swollen joint. Patients with other arthritides and patients with suspected or verified infection were excluded. Patient characteristics are shown in Supplementary Table 1. The procedures were approved by the Ethics Committee of Gothenburg (SYNFLUID study: Dnr 459–18), and all patients gave written informed consent. The concentrations of sPD1 were measured in the paired plasma and synovial fluid samples using enzyme‐linked immunosorbent assay kit (DuoSet, R&D Systems) according to the manufacturer's instructions. No interference by rheumatoid factor was found when patient plasma samples were analysed with different concentrations of HeteroBlock (Omega Biologicals, Inc).

### Isolation of blood CD3+ T Cells

Buffy coats were obtained from healthy blood donors (*n* = 4) anonymously at Sahlgrenska University Hospital, Gothenburg, Sweden, and no ethical approval was required according to the Swedish legislation section code 4§3p SFS 2003:460 (Law on Ethical Testing of Research Relating to People). CD3+ T cells were isolated from RA peripheral blood mononuclear cells (PBMCs) and healthy donor buffy coats using the Pan T cell negative selection bead assay (Miltenyi Biotec, Bergisch Gladbach, Germany) according to the manufacturer's instructions. Purity of CD3+ cells was > 90% as confirmed with flow cytometry.

### Co-culture of fibroblast-like synoviocytes and T cells

Cultured primary RA-FLS or NI-FLS were seeded in 24-well plates at 5 × 10^4 cells per well and left to adhere overnight. Cells were stimulated with 10 ng/mL (final concentration) recombinant TNF (PeproTech, Inc., Rocky Hill, NJ, USA) in Ex-Vivo media supplemented with 0.5% Penicillin–Streptomycin. After 24 h, freshly isolated CD3+ T cells that had been pre-activated with 2 μg/mL anti-CD3 (OKT; eBioscience) for 30 min were added to allogeneic FLS at a 5:1 ratio (T cell:FLS). The final concentration of anti-CD3 in the co-culture was 1 μg/mL. After three days, T cells were carefully removed by repeated washing with PBS. FLS were detached with 0.05% trypsin–EDTA (Life Technologies Inc.), washed in PBS, and resuspended in PBS for further analysis with flow cytometry. Previous studies using human dermal fibroblasts have shown that fibroblasts are poor generators of allogeneic responses [19]. Experiments using cancer-associated fibroblasts have shown that T cell proliferation is similar in allogenic and autologous co-cultures of fibroblasts and T cells [20]. We confirmed these results using FLS.

### Stimulation of fibroblast-like synoviocytes and flow cytometry

FLS cell lines from seven RA patients and seven NI controls were seeded in 24-well plates at 5 × 10^4 cells per well, serum-starved overnight in DMEM with 1% FBS, and then stimulated for 48 hours with the recombinant human proteins IFNγ (10 ng/mL; Thermo Fisher), TNF (10 ng/mL; PeproTech), interleukin-4 (IL-4; 10 ng/mL; PeproTech), or transforming growth factor beta (TGF-β; 10 ng/mL, R&D Systems, Minneapolis, USA). FLS were detached with 0.05% trypsin–EDTA, washed in PBS, and resuspended in PBS for further analysis with flow cytometry. All concentrations refer to final working concentrations.

To examine surface expression of PD-L1 and PD-L2 by flow cytometry, FLS were stained with a 1:2000 dilution of fixable Viability dye eFluor® 581 (eBioscience) for 30 min at room temperature. Unspecific binding of antibodies was blocked using Fc-block (BD Biosciences, New Jersey, USA; 1:100 dilution) for 20 min at 4 °C, before the addition of fluorochrome-conjugated antibodies against PD-L1 (clone 29E.2A3; BioLegend, San Diego, CA, USA) and PD-L2 (clone MIH18; eBioscience). Cells were acquired using a FACSVerse or FACSLyric flow cytometer (BD Biosciences) and analysed using FlowJo software (Tree Star Inc., Ashland, OR, USA). Median fluorescence intensity was used to evaluate the expression levels of PD-L1 and PD-L2 on FLS following stimulation with unstimulated cells as a reference. A detailed gating strategy is presented in Supplementary Fig. 1A-B.

### CRISPR/Cas9 knockout of PD-L1 and PD-L2 in fibroblast-like synoviocytes

Lipofection with Lipofectamine™ CRISPRMAX Cas9 Transfection Reagent (Thermo Fisher, Waltham, MA, USA) was performed according to the manufacturer's instructions. Briefly, FLS cell lines from three RA patients and three NI controls were seeded in 6-well plates at 1 × 10^5 cells per well and were maintained until 70% confluency. Synthetic guide RNA (sgRNA) targeting PD-L1 (CD274, catalogue no. #A35533), PD-L2 (PDCD1LG2, catalogue no. #A35533), or non-target control (catalogue no. #A35526) was mixed with Cas9 nuclease and Cas9 Plus™ reagent in Opti-MEM media (Thermo Fisher). CRISPRMAX™ Reagent diluted in Opti-MEM media was added to the Cas9-sgRNA mix and incubated at room temperature for 7 min. Lastly, the mixture was added directly to cultured cells and incubated at 37 °C with 5% CO_2_ for 3 days before being used for experiments. Successful transfection was confirmed by assessing the surface expression of PD-L1 or PD-L2 on TNF-stimulated cells using flow cytometry as described above, and shown in Supplementary Fig. 2A-C. Following TNF stimulation, knockout FLS had reduced induction of surface PD-L1 or PD-L2, respectively, compared to mock-transfected cells (Supplementary Fig. 3A-B). The optimal time for TNF stimulation to observe differences in surface expression of PD-L1 and PD-L2 was 48 hours (Supplementary Fig. 3C-D).

### Soluble PD1 treatment of TNF-stimulated fibroblast-like synoviocytes

PD-L1-, PD-L2- and mock-transfected FLS cell lines from three RA patients and three NI controls were cultured under five different conditions: (1) Control: unstimulated mock-transfected cells, (2) TNF: TNF-stimulated mock-transfected cells, (3) TNF + sPD1: TNF-stimulated and sPD1-treated mock-transfected cells, (4) No PD-L1 signalling: TNF-stimulated and sPD1-treated PD-L1-transfected cells, and (5) No PD-L2 signalling: TNF-stimulated and sPD1-treated PD-L2-transfected cells. Cells were seeded in 6-well plates at 1.2 × 10^5 cells per well, stimulated with TNF in DMEM containing 1% FBS for 48 hours, and then treated with human PD1 Fc recombinant protein for 24 hours (1 μg/mL; PeproTech, #310–40). There were no visible signs of cell death in the samples at the time of harvest. To exclude potential Fc receptor–mediated artefacts, Fc-blocking experiments were performed prior to sPD1 stimulation, which did not alter IL-6 gene expression responses by qPCR.

### RNA extraction and sequencing of fibroblast-like synoviocytes

From the five different conditions, total RNA was isolated using the RNeasy Micro Kit (Qiagen, Hilden, Germany) according to the manufacturer’s protocol and quantified using the Qubit RNA HS assay (Thermo Fisher). RNA quality was assessed using a NanoDrop One spectrophotometer (Thermo Fisher), reaching A260/280 between 1.99 and 2.18. Extraction of RNA was conducted on the same day and by the same experimenter to avoid batch effects, and sent for sequencing by Xpress Genomics (Stockholm, Sweden). Briefly, automated total RNA sequencing was performed using Xpress-seq total, at a sequencing depth of approximately 30 million reads per sample. Quality control reports were aggregated using MultiQC [21]. Sequenced reads were trimmed using fastp and then aligned to the human reference genome (hg38) using the STAR alignment tool [22, 23]. The number of reads mapped to each gene (gencode v45 gene models) was counted using featureCounts [24].

### Analysis of RNA sequencing data

Pre-processing of count data was performed as previously described [25], using the edgeR and limma/voom R packages (R in RStudio, R Foundation for Statistical Computing, Vienna, Austria). Genes were filtered based on the counts per million (CPM) thresholds corresponding to 10 counts in the smallest library, resulting in a set of 20,495 genes for downstream analysis. Of the total mRNA analyzed, 68.7% were protein-coding and 22.3% long non-coding. Count data were transformed to log_2_-CPM, normalized using the trimmed mean of M-values (TMM) method, and precision-weighted. Genes were re-annotated using the Ensembl BioMart database. Principal component analysis (PCA) was then performed to identify major sources of variation in gene expression.

To compare global transcriptional differences between treatment conditions within RA and NI control samples, we constructed linear models with disease state (RA or NI controls) included as an interaction term, and donor (cell line) as a random effect, using the duplicateCorrelation function in limma. This blocking strategy allows limma to compare treatment-induced fold changes within each cell line rather than variability between cell lines, similar to a paired analysis. The efficacy of this correction was assessed by modelling cell line as a fixed effect in RA and NI control samples separately, performing a PCA, and examining the principal component 1 and 2 of the residual matrix, which contains the variance not captured by cell line. Differential expressions were assessed using an empirical Bayes moderated *t*-test. In the primary analysis, the following comparisons were made: (A) TNF vs control, (B) TNF + sPD1 vs control, and (C) TNF vs TNF + sPD1. The resulting *p*-values were corrected for multiple testing using the Benjamini–Hochberg false discovery rate. Genes were considered differentially expressed if adjusted *p* ≤ 0.05. Reliability of the comparisons was estimated using a bootstrap-based sensitivity analysis as described previously [26]. Briefly, the original linear model was run to obtain reference log_2_ fold-change (log_2_FC) estimates for each gene and comparison. Next, within each group (RA and NI controls), cell lines were repeatedly resampled with replacement, the same linear model refit, and the bootstrap-derived log_2_FC values were compared to the original estimates using Spearman correlation. High correlation (> 0.9) indicates stable and reproducible gene-level rankings, whereas lower correlation (< 0.8) suggests strong dependence on individual samples [26]. In the secondary analysis, “sPD1-responsive” genes were defined by two criteria: (1) TNF + sPD1 required a 50% increase or decrease in gene expression (│log_2_FC│ ≥ 0.585 with unadjusted *p* ≤ 0.05) compared to TNF alone, and (2) No PD-L1 and/or PD-L2 signalling samples required a small or unchanged gene expression compared to TNF (│log_2_FC│ < 0.585 with *p* ≥ 0.5).

### Validation of sPD1-responsive genes identified with RNA sequencing

Complementary DNA (cDNA) was synthesized from the same RNA isolates that were used for RNA-seq analyses, using the High-Capacity cDNA Reverse Transcription Kit (Applied Biosystems, Waltham, USA) according to the manufacturer’s instructions. Expression levels and directionality of selected sPD1-responsive genes were assessed by quantitative real-time PCR (qPCR). Briefly, 10 ng of cDNA was combined with TaqMan Universal PCR Master Mix (Thermo Fisher Scientific, #4369016) and pre-designed TaqMan primer–probe assays (Thermo Fisher Scientific) in a final reaction volume of 10 µL. Amplification signals were measured in technical duplicates using a QuantStudio 6 Pro system (Applied Biosystems). To ensure reliable qPCR quantification, genes with Ct ≥ 30 for TNF-stimulated conditions were excluded. Relative mRNA expression was calculated using the comparative Ct (ΔΔCt) method, with target gene Ct values normalized to hypoxanthine phosphoribosyltransferase 1 (HPRT1) and expressed relative to TNF alone.

### Analysis of single-cell- and bulk RNA-sequencing data from publicly available datasets

Pre-aligned (Drop-seq pipeline) CEL-Seq2 count matrices of filtered reads (UMI ≥ 10) and metadata files from the AMP-RA study were used for analyses of PD-L1 and PD-L2 expression in single cells from established RA synovial tissue samples. Low quality or dead cells, e.g. cells with less than 1000 genes and/or > 25% mitochondrial RNA, were removed followed by normalization and scaling using NormalizeData and ScaleData (R in RStudio). Duplicate cells were removed using scDblFinder. Correction for cell cycle genes was not necessary. Percent PD-L1 and PD-L2 expressing cells were defined as the number of non-zero (count > 0) cells among all cells. Pseudobulk expression values (log_2_-CPM) were compared between lymphocyte-rich and lymphocyte-poor samples, grouping all cells from the same sample and treating each sample as a biological replicate.

Bulk-RNA seq count matrices (GSE89408), consisting of synovial tissue samples from treatment-naïve eRA and established RA patients, were transformed to log_2_-CPM, normalized using the TMM method, and precision-weighted as described above. Gene set variation analysis (GSVA) was performed to detect enrichment of a gene set within the sample population using the GSVA package in R, as previously described [6].

### Statistical analysis

Spearman’s non-parametric test was used to estimate the correlation coefficient between two variables. Correlation strength was determined by the correlation coefficient (r) as weak (0.2 ≤ *r* < 0.4), moderate (0.4 ≤ *r* < 0.6), strong (0.6 ≤ *r* < 0.8), or very strong (*r* ≥ 0.8). Group comparison was performed using Mann–Whitney U test. Pairwise comparisons were performed using the Wilcoxon matched pairs signed rank test, or the Friedman test followed by Dunn’s multiple comparison test. All statistical analyses were done using GraphPad Prism (version 10.2.0, La Jolla, CA, USA). Statistical significance was defined as *p* ≤ 0.05.

## Results

### PD-L1 and PD-L2 gene expression in synovial tissue from RA patients

**Fig. 1:**
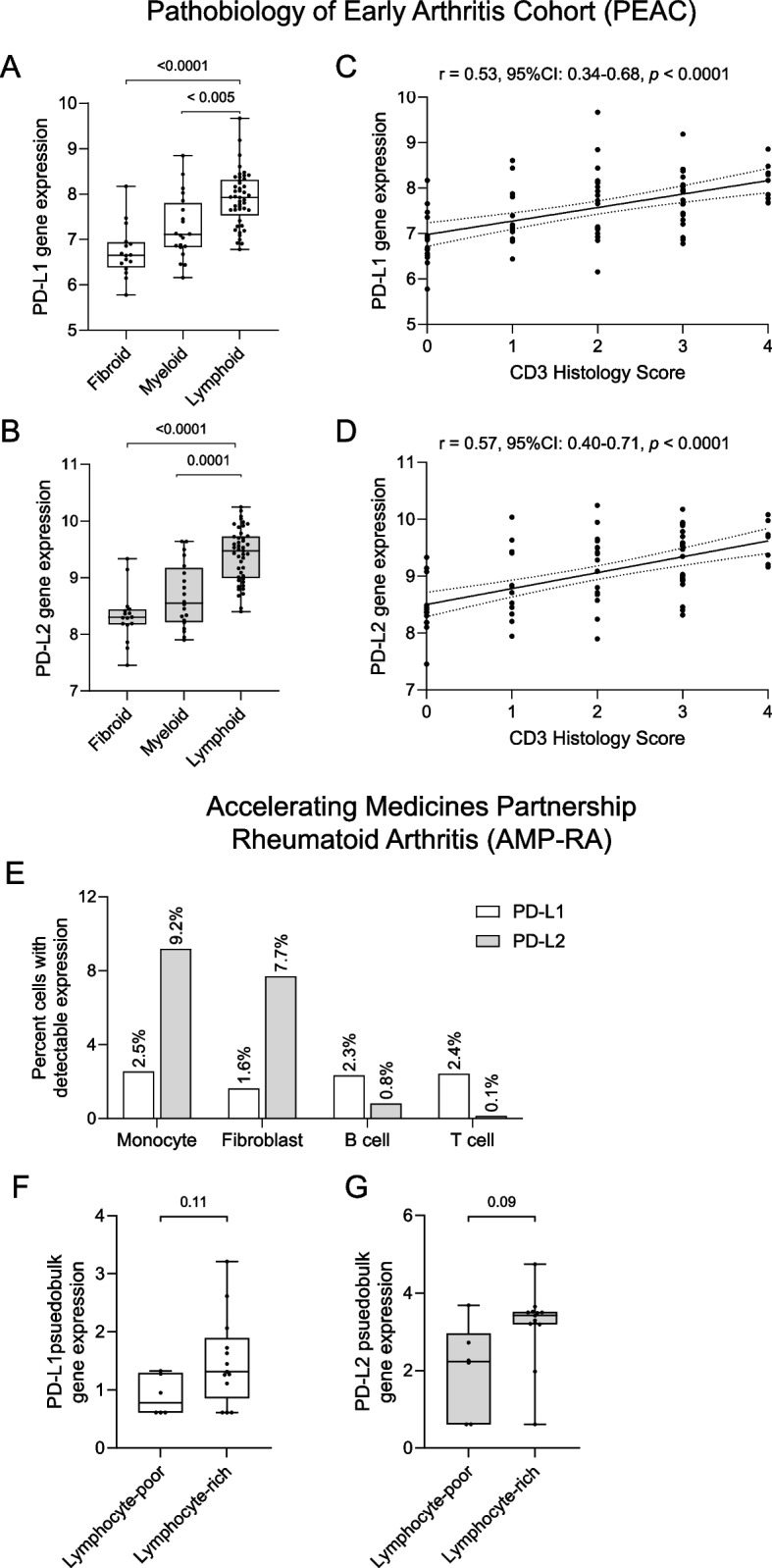
Gene expression of PD-L1 and PD-L2 in synovial tissue from untreated eRA and RA patients. **A**-**D** show data from the PEAC cohort (https://peac.hpc.qmul.ac.uk/). Expression of (**A**) PD-L1 and (**B**) PD-L2 across fibroid (*n* = 16), myeloid (*n* = 20), and lymphoid (*n* = 45) synovial pathotypes from untreated early RA patients. Group comparisons were performed using the Kruskal–Wallis test with Dunn’s post hoc correction. Correlations between synovial gene expression of (**C**) PD-L1 and (**D**) PD-L2 with the CD3 histology score (*n* = 78). Spearman’s correlation coefficients (r) and 95% CI with *p*-value are shown. **E**–**G** shows data from the AMP-RA cohort. **E** Percent cells with detectable expression of PD-L1 or PD-L2. Pseudobulk gene expression of (**F**) PD-L1 and (**G**) PD-L2 in lymphoid-rich and lymphoid-poor synovial tissue samples from established RA patients. Mann–Whitney U-test. CI: confidence interval; PD-L: programmed cell death ligand; RA: rheumatoid arthritis

Publicly available RNA sequencing datasets, such as the PEAC study and the AMP-RA study, enable comparisons of synovial and cell-type-specific gene expression with clinical and histological parameters [1, 18]. Within the PEAC bulk RNA-sequencing dataset, transcript levels of PD-L1 and PD-L2 were significantly higher in treatment-naïve eRA lymphoid synovial tissue compared with myeloid or fibroid synovial pathotypes (Fig. 1A-B). Furthermore, PD-L1 and PD-L2 expression levels correlated with the histological CD3 score (*r* = 0.53, 95% CI: 0.34–0.68; *r* = 0.57, 95% CI: 0.39–0.71, respectively; Fig. 1C-D). A moderate to strong correlation between PD-L1 and PD-L2 transcript levels was observed in the lymphoid and myeloid synovial pathotypes, but not in the fibroid pathotype (Supplementary Fig. 4A). In the AMP-RA single-cell sequencing dataset, PD-L1 was expressed at similar frequencies across monocytes, fibroblasts, B cells, and T cells, whereas monocytes and fibroblasts were the predominant sources of PD-L2 in established RA (Fig. 1E and Supplementary Fig. 4B-D). In pseudobulk analyses aggregating fibroblast transcriptomes for each sample, there was a non-significant trend toward higher PD-L1 and PD-L2 expression in lymphoid-rich compared with lymphoid-poor synovial tissue samples (Fig. 1F-G).

### Cultured primary FLS upregulate PD-L1 and PD-L2 in response to T cell-associated cytokines

**Fig. 2:**
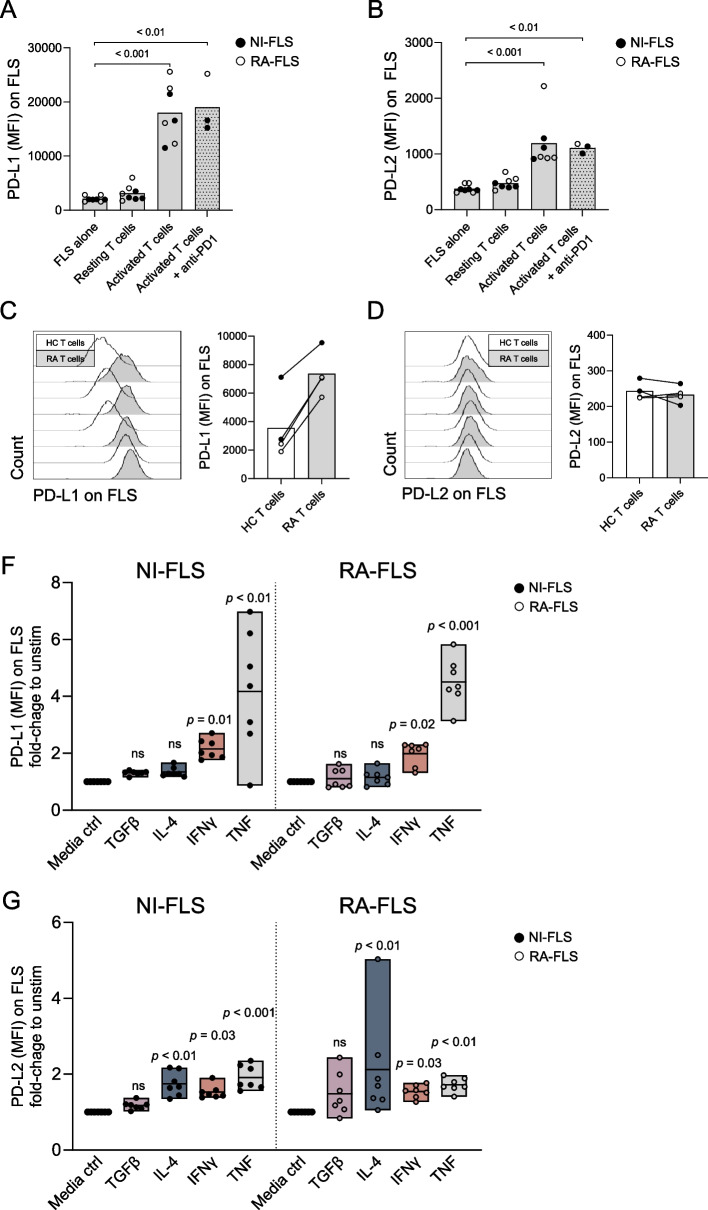
Regulation of PD-L1 and PD-L2 expression on FLS by CD3+ T cells and cytokines. FLS from RA patients (black circles) and NI controls (white circles) were cultured alone, with resting T cells, with anti-CD3-activated T cells, or with anti-PD1 plus activated T cells from HC for 72 hours and the surface expression of (**A**) PD-L1 and (**B**) PD-L2 was analysed by flow cytometry. FLS (RA-FLS in black circles and NI-FLS in white circles) were co-cultured with RA or HC CD3+ T cells, and surface expression of (**C**) PD-L1 and (**D**) PD-L2 was assessed. Expression of (**F**) PD-L1 and (**G**) PD-L2 after 48 hours stimulation with TNF, IFNγ, IL-4, or TGF-β, shown as fold-change relative to unstimulated controls. Bars indicate mean values and circles represent individual FLS cell lines. Statistical comparisons were made using the Kruskal–Wallis test with Dunn’s post hoc test or the Wilcoxon signed-rank test for paired data. FLS: fibroblast-like synoviocytes; HC: healthy controls; NI: non-inflammatory; ns: non-significant; PD-L: programmed cell death ligand; RA: rheumatoid arthritis

Given that PD-L1 and PD-L2 gene expression levels were elevated in lymphoid compared with myeloid and fibroid synovial pathotypes in early RA, we hypothesized that joint tissue-derived FLS would upregulate these ligands in response to T cell infiltration. To test this, PD-L1 and PD-L2 expression were first examined on FLS (from RA and NI controls) cultured without T cells, in the presence of resting T cells, or anti-CD3-activated T cells from HC. As shown in Fig. 2A-B, FLS expressed significantly higher levels of both PD-L1 and PD-L2 in co-culture with activated T cells compared with resting T cells and FLS without T cells. On anti-CD3-activated T cells, PD1 expression was significantly increased compared to resting T cells (Supplementary Fig. 5). However, the addition of anti-PD1 antibodies (Nivolumab) did not affect PD-L1 or PD-L2 expression on FLS induced by activated T cells (Fig. 2A-B), suggesting that neither PD-L1 nor PD-L2 upregulation is dependent on PD1 binding.

We have previously shown that treatment-naïve eRA patients display a distinct profile of circulating Th subtypes compared to age- and sex-matched HC [27]. Therefore, we next measured the surface expression of PD-L1 and PD-L2 on FLS (from RA and NI controls) co-cultured with CD3+ T cells from RA or HC. FLS co-cultured with RA T cells expressed higher PD-L1 than those cultured with HC T cells (Fig. 2C), while PD-L2 expression on FLS was comparable in co-cultures with RA or HC T cells (Fig. 2D).

Finally, we examined whether cytokines associated with different Th subtypes influenced PD-L1 and PD-L2 expression on FLS from RA and NI controls. Stimulation with the pro-inflammatory cytokines IFNγ and TNF increased PD-L1 expression approximately fourfold compared with unstimulated controls (Fig. 2F), whereas the Th2-associated cytokine IL-4 and the immunosuppressive cytokine TGF-β did not. By contrast, PD-L2 expression was upregulated approximately twofold by TNF, IFNγ, and IL-4 but not by TGF-β (Fig. 2G). Notably, RA- and NI-derived FLS responded similarly with respect to PD-L1 and PD-L2 expression in all experimental settings.

In summary, our results show that activated T cells, but not resting T cells, induce PD-L1 and PD-L2 expression on FLS. Moreover, PD-L1 is predominantly regulated by pro-inflammatory cytokines, whereas PD-L2 is upregulated in response to both pro-inflammatory and Th2-associated cytokines.

### Soluble PD1 affects global gene expression in NI-FLS, but not in RA-FLS

**Fig. 3:**
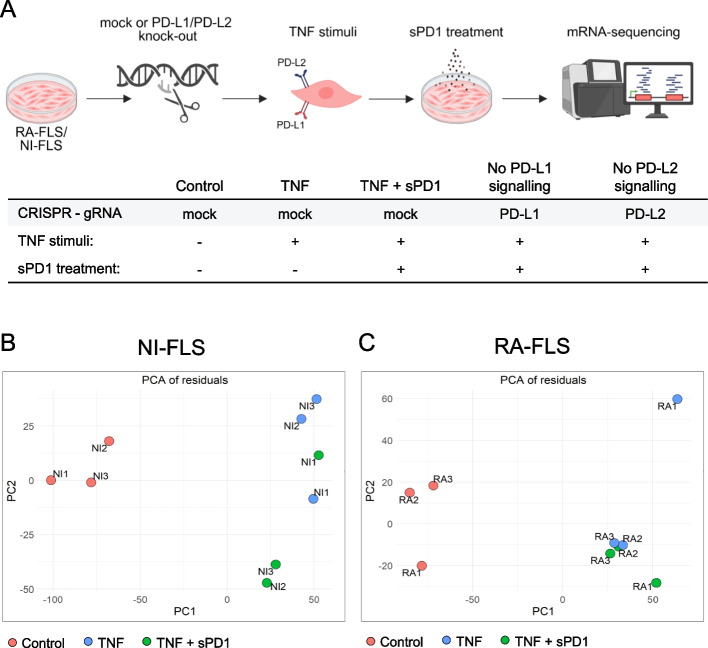
TNF-primed RA-FLS and NI-FLS treated with or without recombinant sPD1. **A** Primary FLS cell lines from three RA patients and three NI controls in passage 3–4 were cultured under five different conditions: (1) unstimulated control (mock-transfected), (2) TNF (mock-transfected), (3) TNF + sPD1 treated (mock-transfected). (4) TNF + sPD1 treated PD-L1 knockout, (5) TNF + sPD1 treated PD-L2 knockout. For the RA and NI groups separately, cell line was modelled as a single fixed effect. **B-C** show PCA plots of the residual matrix, which contains the variance not captured by cell line, for (**B**) NI-FLS and (**C**) RA-FLS under control, TNF, and TNF + sPD1 conditions. FLS: fibroblast-like synoviocytes; NI: non-inflammatory; PCA: principal component analysis; PD-L: programmed cell death ligand; RA: rheumatoid arthritis; sPD1: soluble programmed cell death 1

Most T cells within the RA synovium express high levels of PD1 [16], and sPD1 is elevated in RA synovial fluid compared with paired blood samples (Supplementary Fig. 6). To examine how the crosstalk between T cells and fibroblasts via PD1 to PD-L1 and PD-L2 influences downstream gene expression in FLS, TNF-primed RA- or NI-FLS were treated with recombinant sPD1 and assessed using total mRNA sequencing. The full experimental design is shown in Fig. 3A. We first performed a PCA to obtain an overview of transcriptional changes under different conditions. This analysis confirmed a clear separation between control and TNF-stimulated RA- and NI-FLS, while TNF-stimulated samples with and without sPD1 did not separate (Supplementary Fig. 7 A). PCA further indicated that individual variability and disease state (RA vs NI controls) explained more variance than treatment condition (Supplementary Fig. 7B-C). However, when individual cell lines were modelled as a fixed effect, residual PCA revealed that TNF-stimulated NI-FLS with and without sPD1 began to cluster separately, whereas RA-FLS did not (Fig. 3B-C). Subsequent analyses were therefore performed separately for NI- and RA-FLS, with cell line being included as a random effect.

**Fig. 4:**
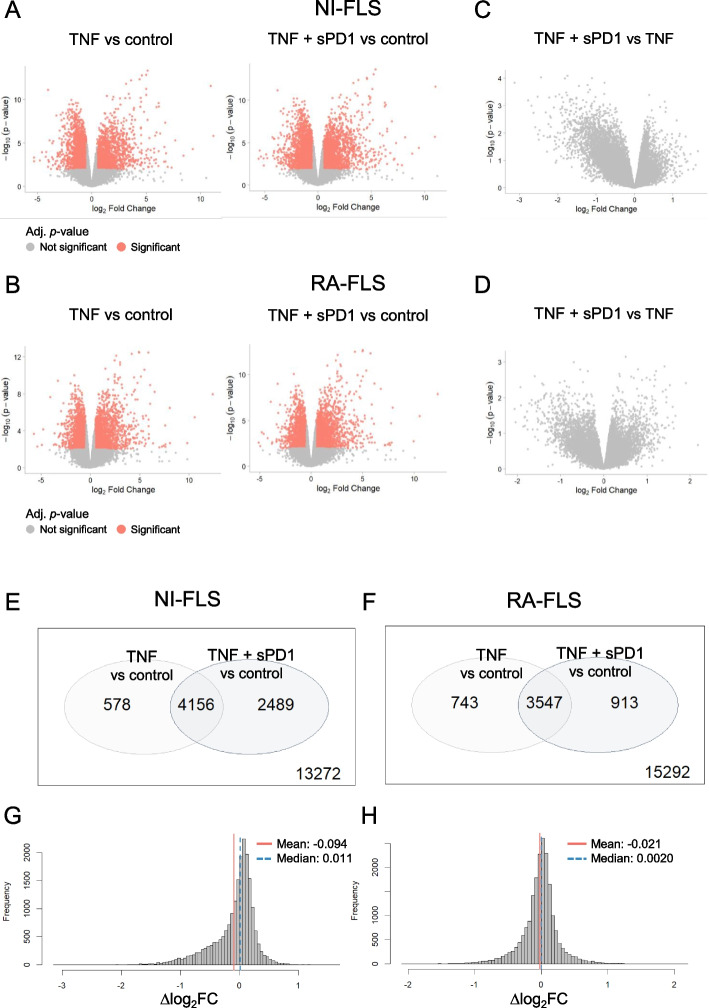
Global transcriptomic effects of sPD1 treatment in TNF-activated RA and NI-FLS. Volcano plots show differentially expressed genes (DEGs; adjusted *p* < 0.05, red circles) for (**A**-**B**) TNF vs control and TNF + sPD1 vs control, and (**C**-**D**) TNF + sPD1 vs TNF for FLS from three RA patients and three NI controls. Venn diagrams show the overlap of DEGs for TNF vs control and TNF + sPD1 vs control in (**E**) NI-FLS and (**F**) RA-FLS. Histogram bar plots show the distribution of ∆log_2_FC values for (**G**) NI-FLS and (**H**) RA-FLS. ∆log_2_FC was calculated for each gene as the log_2_FC value for TNF vs control subtracted from TNF + sPD1 vs control. Red and blue lines indicate median and mean of the frequency distributions. FLS: fibroblast-like synoviocytes; log_2_FC: logarithmic fold-change; NI: non-inflammatory; RA: rheumatoid arthritis; sPD1: soluble programmed cell death 1

Many differentially expressed genes (DEGs) were detected in TNF vs control and in TNF + sPD1 vs control in both NI-FLS and RA-FLS (Fig. 4A-B), but no DEGs were found between TNF + sPD1 vs TNF in either NI-FLS or RA-FLS (Fig. 4C-D), likely due to the low number of samples and the presence of one outlier in each group. Indeed, in bootstrap analysis TNF vs control and TNF + sPD1 vs control demonstrated consistent results (median Spearman correlation > 0.9) across all three cell lines in both NI-FLS and RA-FLS, while TNF + sPD1 vs TNF had a median Spearman correlation of 0.87 for NI-FLS and below 0.8 for RA-FLS (Supplementary Fig. 8), indicating that the signal is dominated by a single cell line and resulting in a high false-positive risk. Focusing on the comparisons TNF vs control and TNF + sPD1 vs control, we found that the overall transcriptional impact of TNF + sPD1 vs control was greater than that of TNF alone, particularly in NI-FLS (Fig. 4E-F). Notably, the number of DEGs identified in the TNF + sPD1 vs control was approximately threefold higher in NI-FLS than in RA-FLS (2489 DEGs in NI-FLS and 913 DEGs in RA-FLS), suggesting that NI-FLS are more transcriptionally responsive to sPD1 engagement than RA-FLS. To assess how sPD1 modifies the TNF-induced transcriptional response, we compared the log_2_FC values of TNF + sPD1 vs control with TNF vs control. In NI-FLS, but not in RA-FLS, this analysis revealed a negatively skewed Δlog_2_FC distribution, indicating an overall dampening of TNF-induced gene expression by sPD1 (Fig. 4G-H). Specifically, 13.8% of genes were dampened (Δlog_2_FC ≤ − 0.5) in NI-FLS following sPD1 treatment, compared with 5.5% in RA-FLS.

In summary, while the lack of reliability due to small sample size and the presence of outliers did not allow for identification of specific TNF-driven genes altered by sPD1 in FLS, our findings indicate that treatment with sPD1 induces greater global transcriptional changes in NI-FLS compared to RA-FLS, suggesting that RA-FLS may have dampened responsiveness to PD1-mediated signals.

### Reverse signalling through PD-L1 and PD-L2 regulates distinct transcriptional profiles in NI-FLS and RA-FLS

**Fig. 5:**
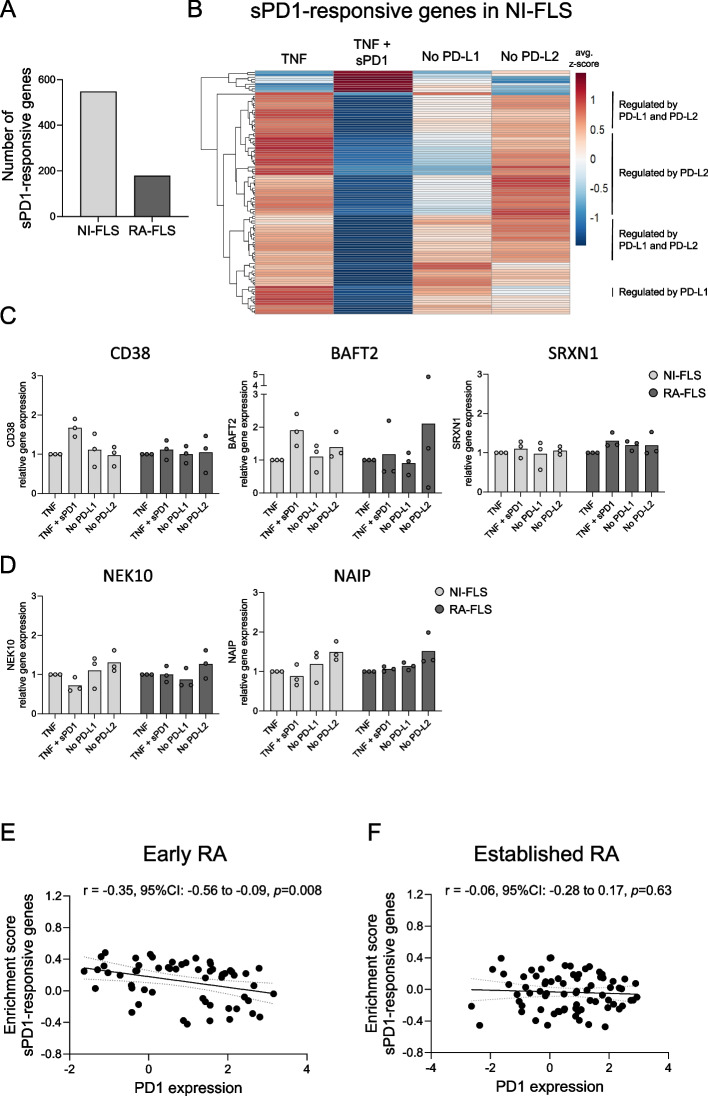
Identification of sPD1-responsive genes in NI-FLS and their enrichment in early and established RA synovial tissue. **A** The number of sPD1-responsive genes in NI- and RA-FLS defined by two criteria: (1) TNF + sPD1 vs TNF: fold change ≥ 50% (|log_2_FC| > 0.585, p ≤ 0.05), and (2) no PD-L1 and/or PD-L2 signalling vs TNF: fold change < 50% (*p* ≥ 0.5). **B** Heatmap with hierarchical clustering of sPD1-responsive genes in mock-transfected NI-FLS stimulated with TNF or TNF + sPD1, and PD-L1 or PD-L2 knockout NI-FLS stimulated with TNF + sPD1. Average z-score was calculated from three biological replicates in each treatment group. **C**-**D** Validation of representative sPD1-responsive genes using qPCR. Relative expression was calculated using the comparative Ct (ΔΔCt) method with TNF alone as reference. CT values were normalized to the housekeeping gene HPRT1. **E–F** show the correlation between enrichment scores of sPD1-responsive genes, identified in NI-FLS, and the PD1 expression levels in (**E**) early and (**F**) established RA synovial tissue samples (GEO database: GSE89408). Spearman’s correlation coefficients (r) and 95% CI with *p*-value are shown. CI: confidence interval; FLS: fibroblast-like synoviocytes; NI: non-inflammatory; PD-L: programmed cell death ligand; RA: rheumatoid arthritis; sPD1: soluble programmed cell death 1

To investigate whether PD-L1 or PD-L2 regulates different genes in FLS, we now included samples where PD-L1 or PD-L2 had been knocked out in our analysis (Fig. 3A). After pre-processing and linear modelling of count data, we sought to identify genes whose expression was increased or decreased due to sPD1 treatment compared to TNF alone but remained unchanged when either PD-L1 or PD-L2 was knocked out on the surface. We identified 547 genes in NI-FLS, but only 178 genes in RA-FLS, that met our filtration criteria, representing potential “sPD1-responsive” genes modulated by PD-L1, PD-L2, or both (Fig. 5A). In NI-FLS, most of these genes had a lower mean expression (avg. z-score) in sPD1 + TNF compared to TNF (Fig. 5B). Notably, for some genes, this effect was reversed when either PD-L1 or PD-L2 had been knocked out, indicating that PD-L1 and PD-L2 may regulate distinct transcriptional responses. To validate our findings, we next performed qPCR of representative sPD1-responsive genes that either increased or decreased following sPD1 treatment compared to TNF alone. In NI-FLS, expression of *CD38* (*cADPR*) and *BATF2* increased (avg. log_2_FC of 0.73 and 0.89) while *NEK10* and *NAIP* decreased (avg. log_2_FC of −0.23 and −0.50) (Fig. 5C-D and Supplementary Fig. 9A). In RA-FLS, no transcriptional changes were observed for these genes. For *SRXN1*, only minor changes in gene expression were detected by qPCR (avg. log_2_FC of 0.12), which may reflect differences in sensitivity between RNA-seq and qPCR. The log_2_FC values obtained from qPCR correlated with those derived from RNA-seq in both NI-FLS and RA-FLS (*r* = 0.65 with *p* = 0.011, and *r* = 0.77 with *p* = 0.001, respectively; Supplementary Fig. 9B), supporting the robustness of the observed transcriptional patterns.

Lastly, we tested whether enrichment of our NI-FLS sPD1-responsive genes correlated with PD1 expression in publicly available RNA-seq dataset (GSE89408) that consists of synovial tissue samples from treatment-naïve eRA and established RA patients. In eRA patients, GSVA enrichment scores of sPD1-responsive genes were negatively correlated with PD1 expression (*r* = −0.35, 95% CI: −0.56 to −0.09; Fig. 5E). In contrast, no correlation was observed in established RA patients (*r* = −0.06, 95% CI: −0.28 to 0.17, *p* = 0.63; Fig. 5F).

Taken together, our findings indicate that PD-L1 and PD-L2 regulate partly distinct gene sets in TNF-stimulated FLS, with RA-FLS displaying more restricted transcriptional responses than NI-FLS. Moreover, the sPD1-responsive genes that were downregulated in NI-FLS correlated negatively with PD1 expression in synovia from eRA, but not established RA patients, which suggests that PD1–ligand interactions are functional in eRA but become altered as the disease progresses.

## Discussion

The mechanisms driving chronic inflammation in RA remain incompletely understood. Investigating T-cell co-inhibitory signalling by FLS within the joint may help clarify why inflammation persists. In this study, we examined how PD-L1 and PD-L2 expression and their downstream signalling on FLS differ between patients with RA and NI controls. In RNA-seq data from treatment-naïve RA patients provided by the PEAC study [1], transcript levels of PD-L1 and PD-L2 were highest in lymphocyte-rich synovial tissue and correlated positively with the histological CD3 score. We show here that PD-L1 and PD-L2 were upregulated on FLS following co-culture with activated CD3+ T cells and upon stimulation with pro-inflammatory and Th2-associated cytokines. Despite distinct cytokine-induced PD-L1 and PD-L2 upregulation, RA- and NI-derived FLS displayed similar expression patterns, suggesting that disease-related differences arise downstream of cytokine signalling. Finally, PD1-ligand reverse signalling modulated TNF-induced gene expression more strongly in NI-FLS than in RA-FLS, supporting that PD1–ligand pathways contribute to immune regulation in health but become altered in RA.

In the dataset from the PEAC study, we found that the PD-L1 and PD-L2 expression correlated with the presence of CD3+ T cells in untreated eRA patients, possibly reflecting an increased demand for local immune regulation during disease development. These results are consistent with previous reports showing elevated PD-L1, PD-L2, and PD1 expression in eRA synovial tissue samples compared to healthy control tissue [9]. Because the PEAC study lacks single-cell resolution, we cannot exclude that other infiltrated PD-L1 and PD-L2 expressing cells, i.e., macrophages and B cells, contribute to the observed increase in PD-L1 and PD-L2 expression. In the AMP-RA dataset, where cell types can be distinguished, we observed a trend toward higher PD-L1 and PD-L2 expression in FLS from lymphocyte-rich compared with lymphocyte-poor established RA tissues. The limited number of patients and the high variability in gene expression between samples likely reduced the statistical power of this comparison. Our in vitro data demonstrate that FLS from established RA upregulate both PD-L1 and PD-L2 in co-culture with activated CD3+ T cells, suggesting a T cell–FLS crosstalk that may drive PD-L1 and PD-L2 expression on FLS in vivo. However, whether crosstalk between FLS and other immune cells, such as macrophages, induces PD-L1 and PD-L2 expression patterns distinct from T cells, and whether such expression occurs in eRA tissue remain to be determined.

A previous study suggested that the PD1 ligands may be unavailable (on the protein level) within the eRA synovium, limiting co-inhibition of PD1+ T cells in RA [9]. Thus, we initially hypothesized that FLS from RA patients would express lower levels of PD-L1 and/or PD-L2 than NI-FLS. However, we show that RA-FLS and NI-FLS, used at similar passage number, exhibited comparable capacities to upregulate these ligands when co-cultured with activated T cells or stimulated with cytokines, indicating that disease-related alterations do not impair the inducibility of PD-L1 and PD-L2. Similar to our findings, skin fibroblasts from patients with vitiligo and healthy donors display comparable PD-L1 induction following IFNγ stimulation [28], suggesting that the capacity of fibroblasts to upregulate PD-L1 may be preserved across autoimmune or inflammation-related conditions. Consistent with previous studies using RA-FLS [7, 29], stimulation with TNF and IFNγ upregulated both PD-L1 and PD-L2 on RA-FLS but also on NI-FLS. In contrast, IL-4 selectively induced PD-L2, identifying IL-4 as a novel inducer of PD-L2 on FLS. This finding is supported by a previous observation investigating mouse macrophages, where IL-4, but not IFNγ plus LPS, induces PD-L2 expression [30]. Together, these data suggest that PD-L1 and PD-L2 are regulated by distinct cytokine pathways but are equally inducible on RA- and NI-FLS. Considering their unique patterns of cytokine regulation and distinct tissue distribution in humans, PD-L1 being enriched in specialized tissues such as the placenta and lymphoid organs, and PD-L2 being more broadly expressed across tissues (Human Protein Atlas proteinatlas.org, [5]), these ligands may serve complementary but distinct roles in maintaining peripheral tolerance.

To our knowledge, this is the first study to demonstrate reverse signalling through PD-L1 and PD-L2 in FLS. As expected, TNF alone induced a broad transcriptional program in FLS, and sPD1 treatment subtly down-modulated this state in NI-FLS, indicating PD1–ligand signalling capacity. This aligns with previous findings in dendritic cells from healthy mice, where sPD1 treatment induced suppressive phenotype characterized by decreased expression of maturation markers and increased IL-10 secretion [31]. In contrast, we found that RA-FLS were less responsive to sPD1. Although not directly investigated here, it is tempting to speculate that chronic inflammation or epigenetic remodelling locks the RA-FLS into the TNF-driven state, limiting further modulation by sPD1. Supporting this hypothesis, a previous study showed that transcriptional profiles of synovial tissue from early and established RA patients resemble those of anti-PD1–treated tumour samples [9]. Our results should, however, be interpreted with caution, as the number of FLS cell lines used to generate the RNA-seq data was small. While the use of a controlled in vitro system focusing on a single cell type together with a paired experimental design increases reliability and partly accounts for inter-donor variability, the small number of samples in each group limits the ability to detect subtle but biologically relevant effects on gene expression, such as those potentially mediated by sPD1 reverse signalling. For this reason, we focused only on global transcriptional patterns rather than individual genes or signalling pathways. Moreover, the TNF-based system used in this study represents a simplified model of RA inflammation, and the complex microenvironment of the RA synovium may therefore reveal distinct effects by sPD1. Others have shown that sPD1 can function as a competitive decoy by disrupting PD1–ligand interactions between PD1- and PD-L1-expressing immune cells [13]. However, in our experimental system, sPD1 was applied to FLS monocultures in the absence of PD1–expressing immune cells. Under these conditions, disruption of the PD1–ligand interaction is unlikely to account for the observed transcriptional changes. Instead, our data support a ligand-dependent effect as knockout of PD-L1 and PD-L2 in FLS reversed some transcriptional responses to sPD1, indicating that these ligands are required for the observed effects. Future studies are needed to define specific pathways regulated by PD-L1 and PD-L2 reverse signalling in FLS, and to validate sPD1-induced effects at the molecular and/or functional level.

The importance of functional PD1 signalling in RA has recently become evident. In patients with RA, anti-PD1 therapy for cancer triggers disease flares in approximately half of treated individuals [32]. A systematic review further demonstrated that the flare-risk is higher in RA patients treated with anti-PD1 than in those with other autoimmune disorders [33]. Conversely, treatment with a PD1 agonist (peresolimab) significantly reduced disease activity at week 12 among patients with active RA who had previously shown inadequate responses to biological disease-modifying antirheumatic drugs, compared with placebo in a phase 2 randomized controlled trial [34]. These findings further support that PD1-mediated immune suppression is functionally impaired established RA. One possible explanation for this is that sPD1 might block the PD1-ligand signalling in RA and arthritic mice models [13, 35], limiting the immunosuppressive capacity of PD1 [12, 35]. Our data adds that sPD1-induced reverse signalling occurs in NI-FLS but is reduced in established RA-FLS. Moreover, genes downregulated by sPD1 in NI-FLS were negatively correlated with PD1 expression in synovial tissue from eRA patients but not in established RA. This implies that disruption of PD1-mediated signalling likely arises at later stages of disease progression, under chronic inflammatory conditions.

## Conclusion

In conclusion, our findings indicate that RA-related alterations do not impair the cytokine-induced expression of PD-L1 and PD-L2 on FLS. However, while PD1–ligand interactions appear functionally active in early disease, they become progressively altered under chronic inflammatory conditions, potentially contributing to the loss of immune regulation in established RA. Therapeutic strategies that restore or enhance PD1 signalling may therefore offer a promising approach in established RA.

## Supplementary Information

Supplementary Material 1.

## Data Availability

The datasets used and/or analysed during the current study are available from the corresponding author on reasonable request.

## Abbreviations

ACR: American College of Rheumatology
APCs: Antigen-presenting cells
CPM: Counts per million
DEG: Differentially expressed genes
DMEM: Dulbecco’s modified Eagle’s medium
EULAR: European Alliance of Associations for Rheumatology
eRA: Early rheumatoid arthritis
FBS: Fetal bovine serum
FC: Fold-change
FLS: Fibroblast-like synoviocytes
GSVA: Gene set variation analysis
HC: Healthy controls
IFNγ: Interferon gamma
IL: Interleukin
NI: Non-inflammatory
PBMCs: Peripheral blood mononuclear cells
PBS: Phosphate-buffered saline
PCA: Principal component analysis
PD1: Programmed cell death-1
PD-L1: Programmed cell death-ligand 1
PD-L2: Programmed cell death-ligand 2
RA: Rheumatoid arthritis
sgRNA: Synthetic guide RNA
sPD1: Soluble programmed cell death-1
TGF-β: Transforming growth factor beta
Th: T-helper
TMM: Trimmed mean of M-values
TNF: Tumour necrosis factor

## References

[1] Lewis MJ, Barnes MR, Blighe K, Goldmann K, Rana S, Hackney JA, et al. Molecular portraits of early Rheumatoid Arthritis identify clinical and treatment response phenotypes. Cell Rep. 2019;28(9):2455-2470.e2455. 31461658 10.1016/j.celrep.2019.07.091 PMC6718830

[2] Aldridge J, Ekwall AH, Mark L, Bergström B, Andersson K, Gjertsson I, et al. T helper cells in synovial fluid of patients with Rheumatoid Arthritis primarily have a Th1 and a CXCR3(+)Th2 phenotype. Arthritis Res Ther. 2020;22(1):245. 33066816 10.1186/s13075-020-02349-y PMC7566124

[3] Tran CN, Lundy SK, White PT, Endres JL, Motyl CD, Gupta R, et al. Molecular interactions between T cells and fibroblast-like synoviocytes: role of membrane tumor necrosis factor-alpha on cytokine-activated T cells. Am J Pathol. 2007;171(5):1588–98. 17823284 10.2353/ajpath.2007.070004 PMC2043519

[4] Køster D, Egedal JH, Lomholt S, Hvid M, Jakobsen MR, Müller-Ladner U, et al. Phenotypic and functional characterization of synovial fluid-derived fibroblast-like synoviocytes in Rheumatoid Arthritis. Sci Rep. 2021;11(1):22168. 34772990 10.1038/s41598-021-01692-7 PMC8590001

[5] Uhlén M, Fagerberg L, Hallström BM, Lindskog C, Oksvold P, Mardinoglu A, Sivertsson Å, Kampf C, Sjöstedt E, Asplund A et al: Proteomics. Tissue-based map of the human proteome. Science. 2015;347(6220):1260419. 10.1126/science.1260419 25613900

[6] Pedersen K, Nielsen MA, Juul-Madsen K, Hvid M, Deleuran B, Greisen SR. Galectin-3 interacts with PD-1 and counteracts the PD-1 pathway-driven regulation of T cell and osteoclast activity in Rheumatoid Arthritis. Scand J Immunol. 2023;97(2):e13245. 36537046 10.1111/sji.13245 PMC10078345

[7] Greisen SR, Kragstrup TW, Thomsen JS, Hansen AS, Krishnamurthy A, Hørslev-Petersen K, et al. Programmed death ligand 2 - a link between inflammation and bone loss in Rheumatoid Arthritis. J Transl Autoimmun. 2020;3:100028. 32743513 10.1016/j.jtauto.2019.100028 PMC7388353

[8] Brown JA, Dorfman DM, Ma FR, Sullivan EL, Munoz O, Wood CR, et al. Blockade of programmed death-1 ligands on dendritic cells enhances T cell activation and cytokine production. J Immunol. 2003;170(3):1257–66. 12538684 10.4049/jimmunol.170.3.1257

[9] Guo Y, Walsh AM, Canavan M, Wechalekar MD, Cole S, Yin X, et al. Immune checkpoint inhibitor PD-1 pathway is down-regulated in synovium at various stages of Rheumatoid Arthritis disease progression. PLoS ONE. 2018;13(2):e0192704. 29489833 10.1371/journal.pone.0192704 PMC5831027

[10] Raza K, Falciani F, Curnow SJ, Ross EJ, Lee CY, Akbar AN, et al. Early Rheumatoid Arthritis is characterized by a distinct and transient synovial fluid cytokine profile of T cell and stromal cell origin. Arthritis Res Ther. 2005;7(4):R784-795. 15987480 10.1186/ar1733 PMC1175027

[11] Latchman Y, Wood CR, Chernova T, Chaudhary D, Borde M, Chernova I, et al. PD-L2 is a second ligand for PD-1 and inhibits T cell activation. Nat Immunol. 2001;2(3):261–8. 11224527 10.1038/85330

[12] Raptopoulou AP, Bertsias G, Makrygiannakis D, Verginis P, Kritikos I, Tzardi M, et al. The programmed death 1/programmed death ligand 1 inhibitory pathway is up-regulated in rheumatoid synovium and regulates peripheral T cell responses in human and murine arthritis. Arthritis Rheum. 2010;62(7):1870–80. 20506224 10.1002/art.27500

[13] Bommarito D, Hall C, Taams LS, Corrigall VM. Inflammatory cytokines compromise programmed cell death-1 (PD-1)-mediated T cell suppression in inflammatory arthritis through up-regulation of soluble PD-1. Clin Exp Immunol. 2017;188(3):455–66. 28245522 10.1111/cei.12949 PMC5422858

[14] Gato-Cañas M, Zuazo M, Arasanz H, Ibañez-Vea M, Lorenzo L, Fernandez-Hinojal G, et al. PDL1 signals through conserved sequence motifs to overcome interferon-mediated cytotoxicity. Cell Rep. 2017;20(8):1818–29. 28834746 10.1016/j.celrep.2017.07.075

[15] Jalali S, Price-Troska T, Bothun C, Villasboas J, Kim HJ, Yang ZZ, et al. Reverse signaling via PD-L1 supports malignant cell growth and survival in classical Hodgkin lymphoma. Blood Cancer J. 2019;9(3):22. 30783096 10.1038/s41408-019-0185-9 PMC6381098

[16] Rao DA, Gurish MF, Marshall JL, Slowikowski K, Fonseka CY, Liu Y, et al. Pathologically expanded peripheral T helper cell subset drives B cells in rheumatoid arthritis. Nature. 2017;542(7639):110–4. 28150777 10.1038/nature20810 PMC5349321

[17] Jiang TT, Martinov T, Xin L, Kinder JM, Spanier JA, Fife BT, et al. Programmed death-1 culls peripheral accumulation of high-affinity autoreactive CD4 T cells to protect against autoimmunity. Cell Rep. 2016;17(7):1783–94. 27829150 10.1016/j.celrep.2016.10.042 PMC5108556

[18] Zhang F, Wei K, Slowikowski K, Fonseka CY, Rao DA, Kelly S, et al. Defining inflammatory cell states in rheumatoid arthritis joint synovial tissues by integrating single-cell transcriptomics and mass cytometry. Nat Immunol. 2019;20(7):928–42. 31061532 10.1038/s41590-019-0378-1 PMC6602051

[19] Geppert TD, Lipsky PE. Antigen presentation by interferon-gamma-treated endothelial cells and fibroblasts: differential ability to function as antigen-presenting cells despite comparable Ia expression. J Immunol. 1985;135(6):3750–62. 3934267

[20] Gorchs L, Fernández Moro C, Bankhead P, Kern KP, Sadeak I, Meng Q, et al. Human pancreatic carcinoma-associated fibroblasts promote expression of co-inhibitory markers on CD4(+) and CD8(+) T-cells. Front Immunol. 2019;10:847. 31068935 10.3389/fimmu.2019.00847 PMC6491453

[21] Ewels P, Magnusson M, Lundin S, Käller M. MultiQC: summarize analysis results for multiple tools and samples in a single report. Bioinformatics. 2016;32(19):3047–8. 27312411 10.1093/bioinformatics/btw354 PMC5039924

[22] Dobin A, Davis CA, Schlesinger F, Drenkow J, Zaleski C, Jha S, et al. STAR: ultrafast universal RNA-seq aligner. Bioinformatics. 2013;29(1):15–21. 23104886 10.1093/bioinformatics/bts635 PMC3530905

[23] Chen S, Zhou Y, Chen Y, Gu J. fastp: an ultra-fast all-in-one FASTQ preprocessor. Bioinformatics. 2018;34(17):i884–90. 30423086 10.1093/bioinformatics/bty560 PMC6129281

[24] Liao Y, Smyth GK, Shi W. featureCounts: an efficient general purpose program for assigning sequence reads to genomic features. Bioinformatics. 2014;30(7):923–30. 24227677 10.1093/bioinformatics/btt656

[25] Law CW, Alhamdoosh M, Su S, Dong X, Tian L, Smyth GK, Ritchie ME. RNA-seq analysis is easy as 1-2-3 with limma, Glimma and edgeR. F1000Res. 2016;5:1408. 10.12688/f1000research.9005.1 PMC4937821 27441086

[26] Degen PM, Medo M. Replicability of bulk RNA-Seq differential expression and enrichment analysis results for small cohort sizes. PLoS Comput Biol. 2025;21(5):e1011630. 40324149 10.1371/journal.pcbi.1011630 PMC12077797

[27] Pandya JM, Lundell AC, Hallström M, Andersson K, Nordström I, Rudin A. Circulating T helper and T regulatory subsets in untreated early rheumatoid arthritis and healthy control subjects. J Leukoc Biol. 2016;100(4):823–33. 27190305 10.1189/jlb.5A0116-025R

[28] Willemsen M, Krebbers G, Tjin EPM, Willemsen KJ, Louis A, Konijn VAL, et al. IFN-γ-induced PD-L1 expression on human melanocytes is impaired in vitiligo. Exp Dermatol. 2022;31(4):556–66. 34758170 10.1111/exd.14500

[29] Greisen SR, Kragstrup TW, Thomsen JS, Hørslev-Pedersen K, Hetland ML, Stengaard-Pedersen K, et al. The Programmed Death-1 Pathway counter-regulates inflammation-induced osteoclast activity in clinical and experimental settings. Front Immunol. 2022;13:773946. 35356000 10.3389/fimmu.2022.773946 PMC8959817

[30] Loke P, Allison JP. PD-L1 and PD-L2 are differentially regulated by Th1 and Th2 cells. Proc Natl Acad Sci U S A. 2003;100(9):5336–41. 12697896 10.1073/pnas.0931259100 PMC154346

[31] Kuipers H, Muskens F, Willart M, Hijdra D, van Assema FB, Coyle AJ, et al. Contribution of the PD-1 ligands/PD-1 signaling pathway to dendritic cell-mediated CD4+ T cell activation. Eur J Immunol. 2006;36(9):2472–82. 16917960 10.1002/eji.200635978

[32] McCarter KR, Arabelovic S, Wang X, Wolfgang T, Yoshida K, Qian G, et al. Immunomodulator use, risk factors and management of flares, and mortality for patients with pre-existing rheumatoid arthritis after immune checkpoint inhibitors for cancer. Semin Arthritis Rheum. 2024;64:152335. 38100899 10.1016/j.semarthrit.2023.152335 PMC10842881

[33] Liu X, Li S, Ke L, Cui H. Immune checkpoint inhibitors in cancer patients with rheumatologic preexisting autoimmune diseases: a systematic review and meta-analysis. BMC Cancer. 2024;24(1):490. 38632528 10.1186/s12885-024-12256-z PMC11025164

[34] Tuttle J, Drescher E, Simón-Campos JA, Emery P, Greenwald M, Kivitz A, et al. A phase 2 trial of Peresolimab for adults with rheumatoid arthritis. N Engl J Med. 2023;388(20):1853–62. 37195941 10.1056/NEJMoa2209856

[35] Liu C, Jiang J, Gao L, Wang X, Hu X, Wu M, et al. Soluble PD-1 aggravates progression of collagen-induced arthritis through Th1 and Th17 pathways. Arthritis Res Ther. 2015;17:340. 26608464 10.1186/s13075-015-0859-z PMC4659197

