# Supplementary material for: Cytokine-induced PD-L1 and PD-L2 expression is preserved but reverse signalling is altered in rheumatoid arthritis fibroblast-like synoviocytes
Source: Arthritis Res Ther. 2026 Jun 2;28:125. doi: 10.1186/s13075-026-03841-7 (PMC13231578; doi:10.1186/s13075-026-03841-7)
Supplement: Supplementary file 1 — Supplementary Material 1. [file 13075_2026_3841_MOESM1_ESM.pdf]

Supplementary files for:

Cytokine-Induced PD-L1 and PD-L2 Expression is Preserved but Reverse Signalling is Altered in Rheumatoid Arthritis Fibroblast-Like Synoviocytes

Tilia Selldén*, Anna-Karin Hultgård Ekwall, Georgios Chatziagorou, Anna-Carin Lundell, and Anna Rudin

*Correspondence:
Tilia Selldén
Department of Rheumatology and Inflammation Research, Sahlgrenska Academy at University of Gothenburg, Box 480, S-405 30, Gothenburg, Sweden
E-mail: tilia.sellden@gu.se; Tel: +46 761068907

Supplementary Table 1

Supplementary Table 1: Characteristics of RA patients

**Donation**	**Synovial tissue (n = 7)**	**Blood and synovial fluid (n = 13)ǂ**
Age [a]	66 (27-72)*	64 (34-79)
Female sex, n (%)	6 (85)	10 (71)
Disease duration, years [a,b]	13 (1-22)**	3 (0-17)**
ACPA+ and/or RF+, n (%) [c,d]	3 (43)	11 (79)

[a] Median and range

[b] Retrospective patient-reported pain in the joints before RA diagnosis

[c] Patients with ACPA levels ≥ 20 IU/ml are considered ACPA+

[d] Patients with RF levels ≥ 20 IU/ml are considered RF+

* Missing data for one patient, ** Missing data for four patients

ǂ Twelve patients had paired blood and synovial fluid

Supplementary Table 2

Supplementary Table 2: Characteristics of non-inflammatory and healthy controls

**Donation**	**Synovial tissue (n = 7)**	**Blood for T cell isolation (n = 4)**
Age [a]	43 (25-70)	N/A
Female sex, n (%)	4 (57)	N/A

[a] Median and range

Supplementary Figure 1

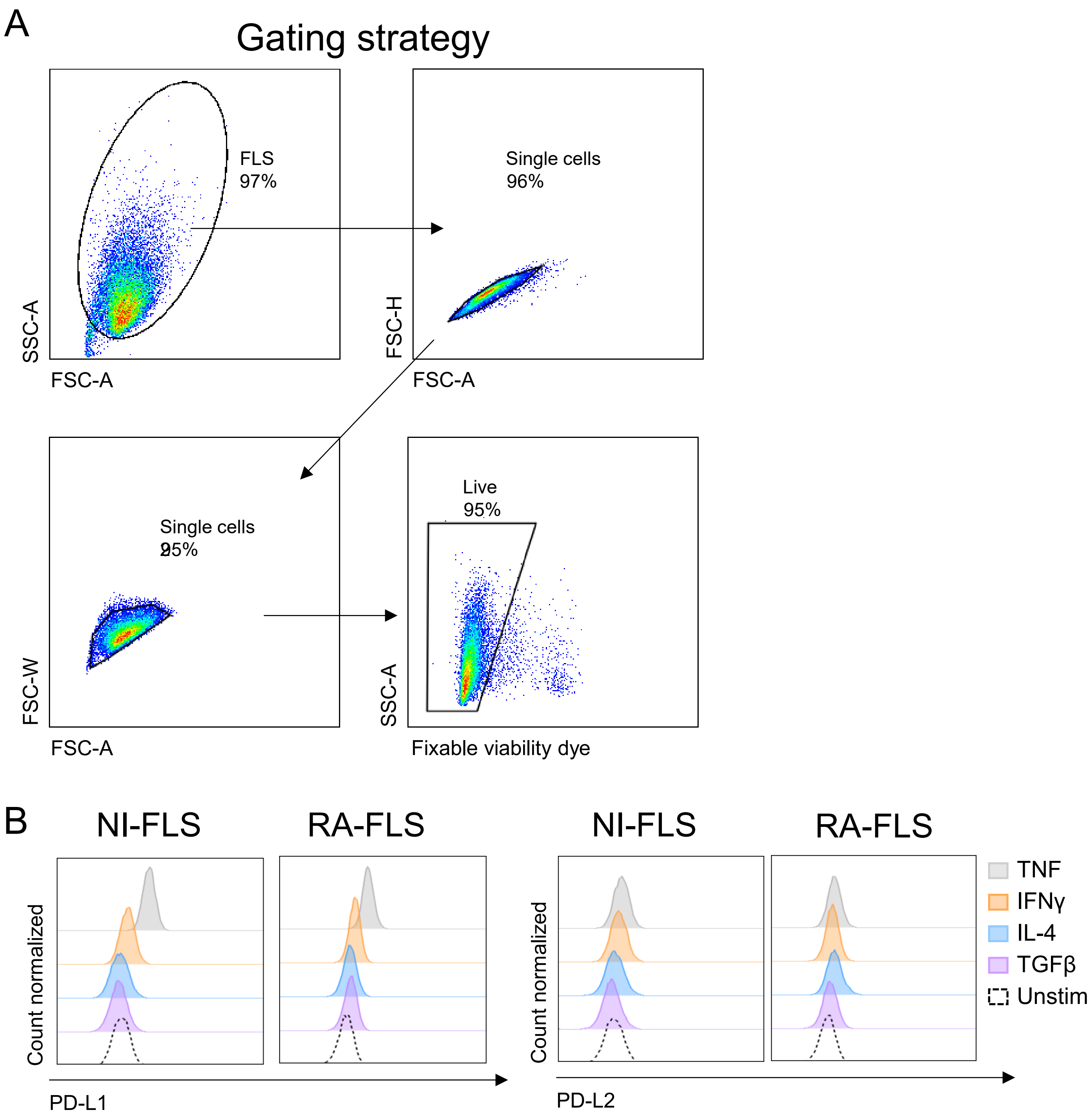

$$\text{Relative expression} = \frac{\text{MFI(stimulated)}}{\text{MFI(unstim)}}$$

Supplementary Fig 1. Flow cytometry gating strategy for assessing PD-L1 and PD-L2 expression on fibroblast-like synoviocytes (FLS). **(A)** Representative flow cytometric dot plots for isolation of viable single cells. **(B)** Representative histogram plots of FLS derived from non-inflammatory (NI) controls or RA patients that was stimulated with TNF, IFNγ, IL-4, or TGFβ for 48 hours. Relative expression was calculated as the median fluorescence intensity (MFI) value of the stimulated sample relative to MFI of unstimulated control.

Supplementary Figure 2

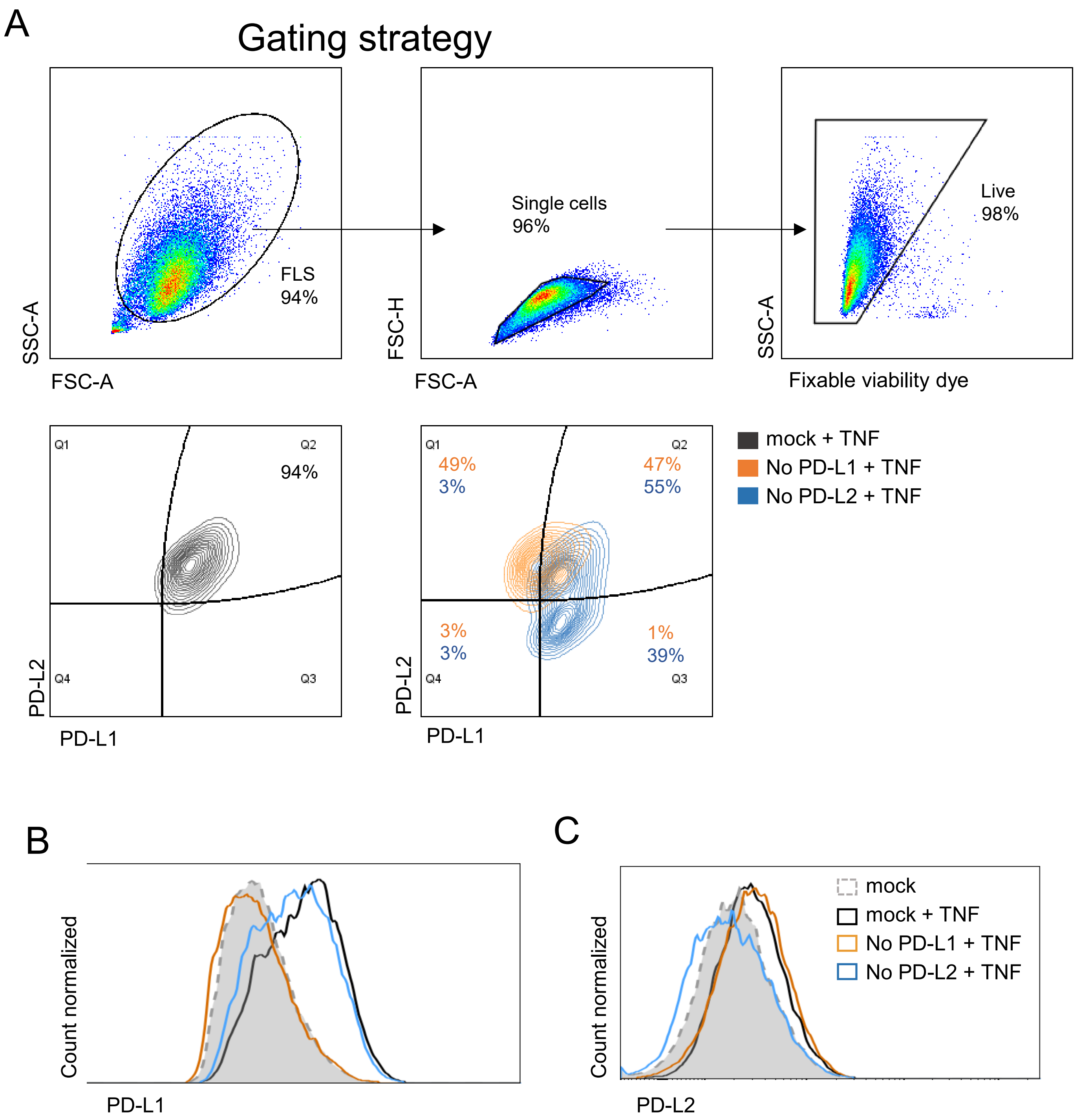

Supplementary Fig 2. Confirmation of PD-L1 and PD-L2 knock-out on fibroblast-like synoviocytes (FLS). **(A)** Flow cytometry gating strategy for assessing PD-L1 and PD-L2 expression on TNF-stimulated (mock-transfected) (black), TNF-stimulated with PD-L1 knock-out (orange), and TNF-stimulated with PD-L2 knock-out (blue) FLS. **(B-C)** Representative histogram plots of PD-L1 and PD-L2 expression on unstimulated (mock-transfected) FLS, TNF-stimulated (mock-transfected), TNF-stimulated with PD-L1 knock-out, and TNF-stimulated with PD-L2 knock-out.

Supplementary Figure 3

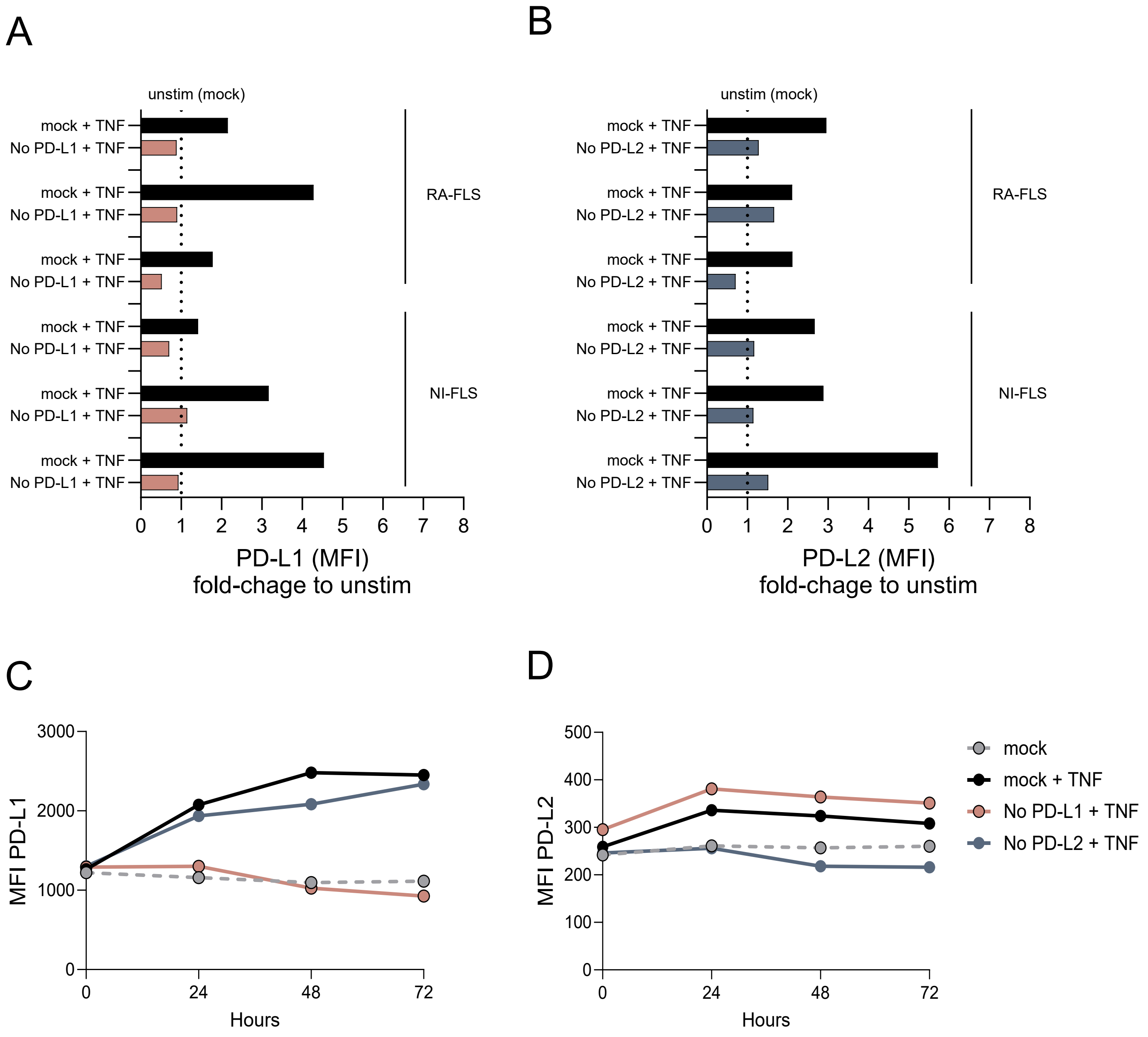

Supplementary Fig 3. (A-B) PD-L1 and PD-L2 expression in knock-out RA-fibroblast-like synoviocytes (FLS) (n = 3) and non-inflammatory (NI)-FLS (n = 3), calculated as the fold-change median fluorescence intensity (MFI) between the stimulated sample and unstimulated control. **(C-D)** Time course of PD-L1 and PD-L2 expression on conditioned FLS. Mock = mock-transfected cells, No PD-L1 = PD-L1-transfected cells, No PD-L2 = PD-L2-transfected cells.

Supplementary Figure 4

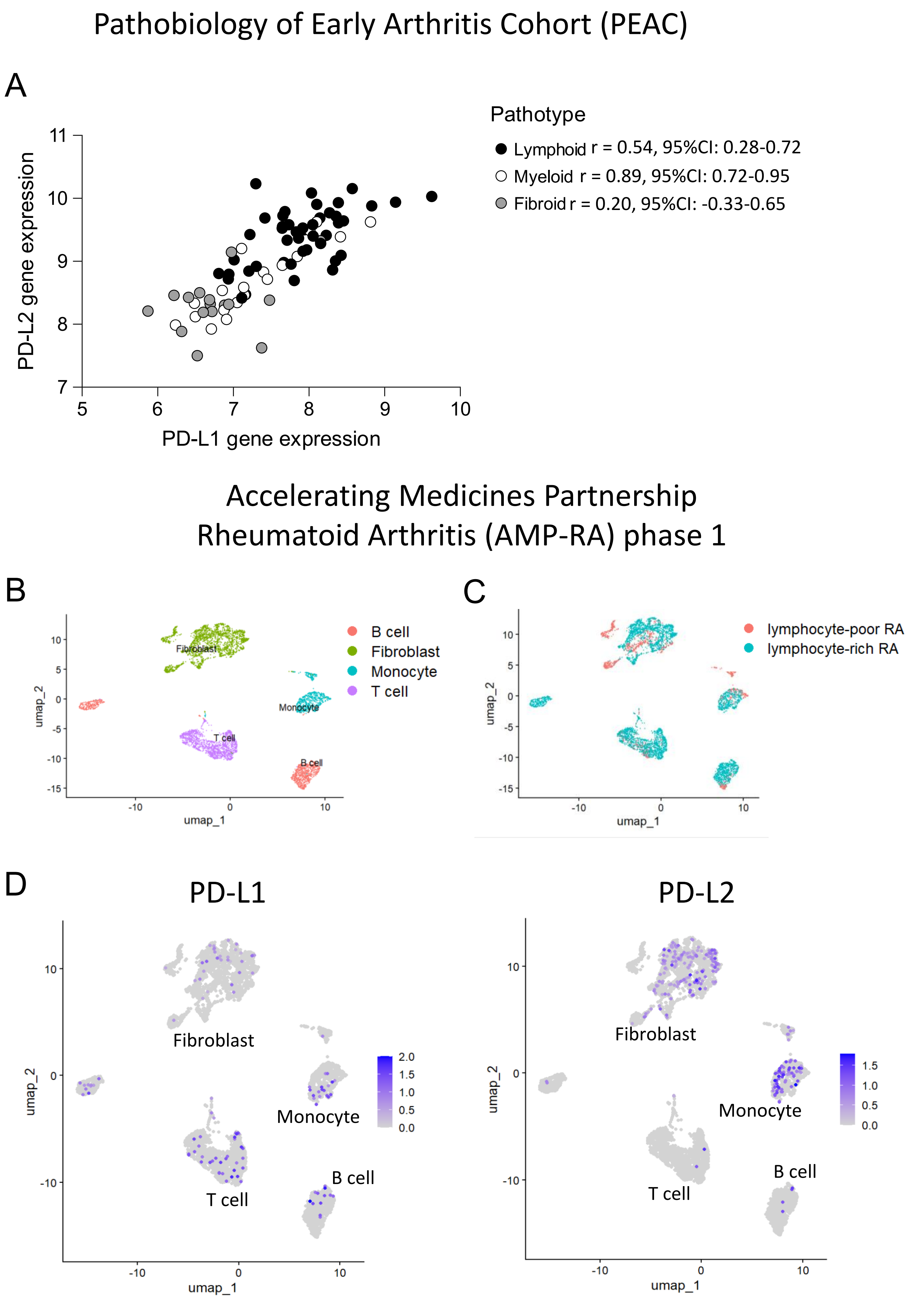

Supplementary Fig 4. (A) shows data from the PEAC cohort. Correlations between PD-L1 and PD-L2 expression within each respective synovial pathotype. B-D shows data from the AMP-RA cohort. UMAP plots showing cell clustering and coloured by **(B)** cell type, **(C)** pathotype or **(D)** PD-L1 and PD-L2 expression.

Supplementary Figure 5

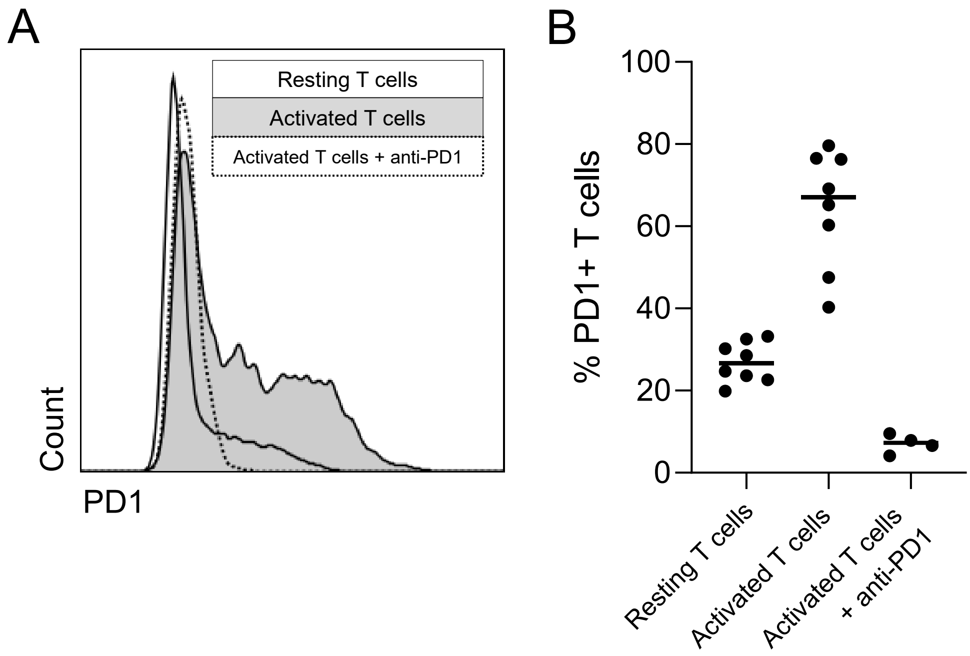

Supplementary Fig 5. Expression of programmed cell death 1 (PD1) on CD3+ T cells. **(A)** Representative histogram plot for assessing PD1 expression by flow cytometry. **(B)** Percentage of PD1+ T cells in resting condition, after stimulation with anti-CD3, and after anti-CD3 + anti-PD1 (nivolumab) treatment.

Supplementary Figure 6

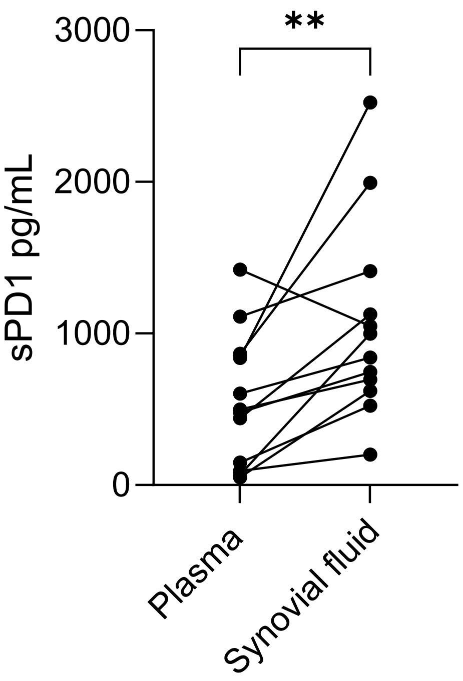

Supplementary Fig 6. Levels of soluble programmed cell death 1 (sPD1) in paired plasma and synovial fluid samples of established RA patients (n = 12). sPD1 levels were measured with enzyme-linked immunosorbent assay.

Supplementary Figure 7

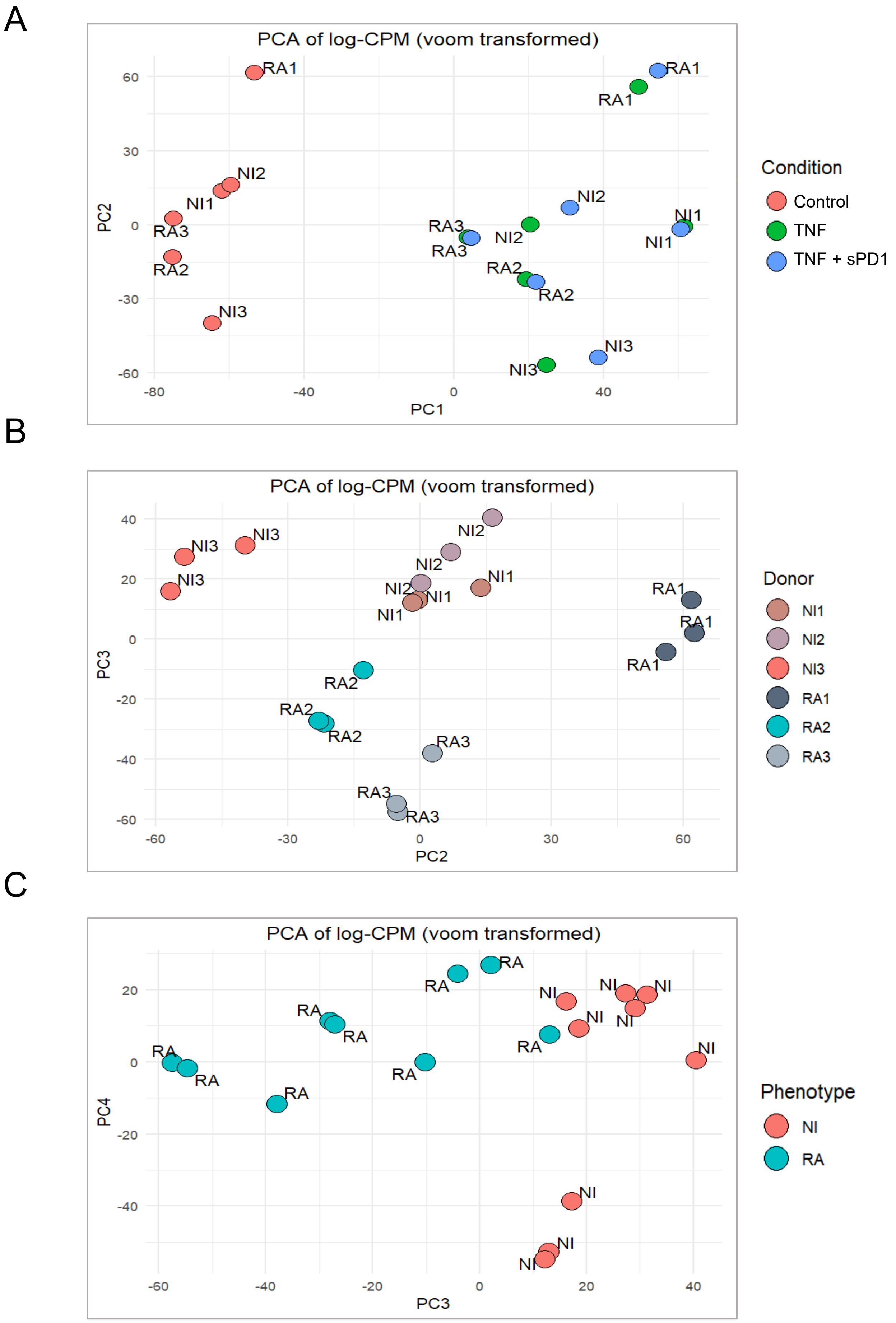

Supplementary Fig 7. Assessment of potential sources of variability within the RNA-seq data. Principal component analysis (PCA) plots of **(A)** PC1 and PC2 (color indicates treatment condition), **(B)** PC2 and PC3 (color indicates donor from which the cells were derived), and **(C)** PC3 and PC4 (color indicates disease state/phenotype). Of the total mRNA analyzed 68.7% were protein-coding and 22.3% long non-coding.

Supplementary Figure 8

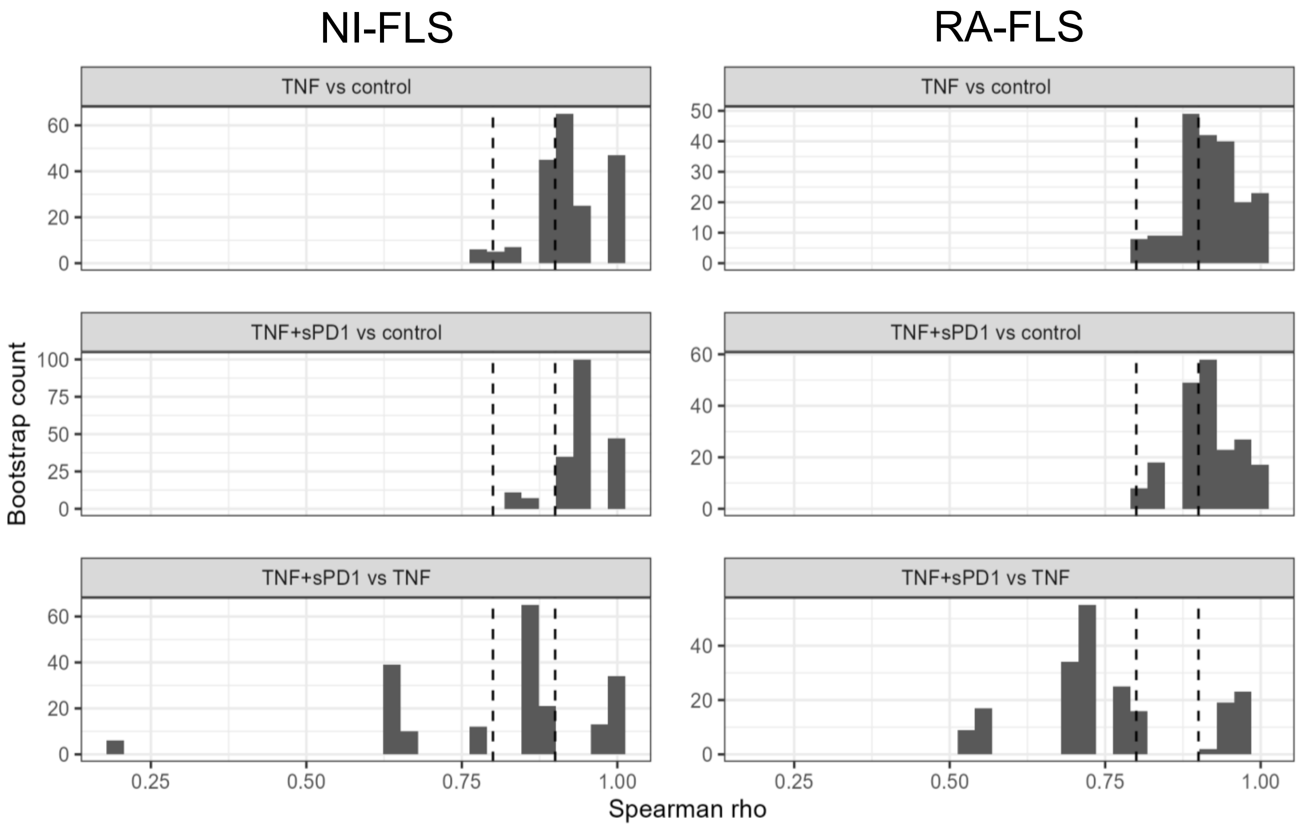

Comparison	Median Spearman correlation (ρ)
NI-FLS:	
TNF vs control	0.915
TNF + sPD1 vs control	0.941
TNF + sPD1 vs TNF	0.869
RA-FLS:	
TNF vs control	0.926
TNF + sPD1 vs control	0.918
TNF + sPD1 vs TNF	0.723

Supplementary Fig 8. Bootstrap analysis of RNA-seq differential expression reliability. For each comparison, histograms show the distribution of Spearman correlations between logFC estimates from the original analysis and from bootstrap-resampled datasets (B = 200). Differential expression was re-computed using limma–voom with duplicateCorrelation, resampling cell lines with replacement within each group (NI and RA). Dashed lines indicate reproducibility thresholds (ρ = 0.8 and 0.9). Higher correlations (> 0.9) indicate stable and reproducible gene-level rankings.

Supplementary Figure 9

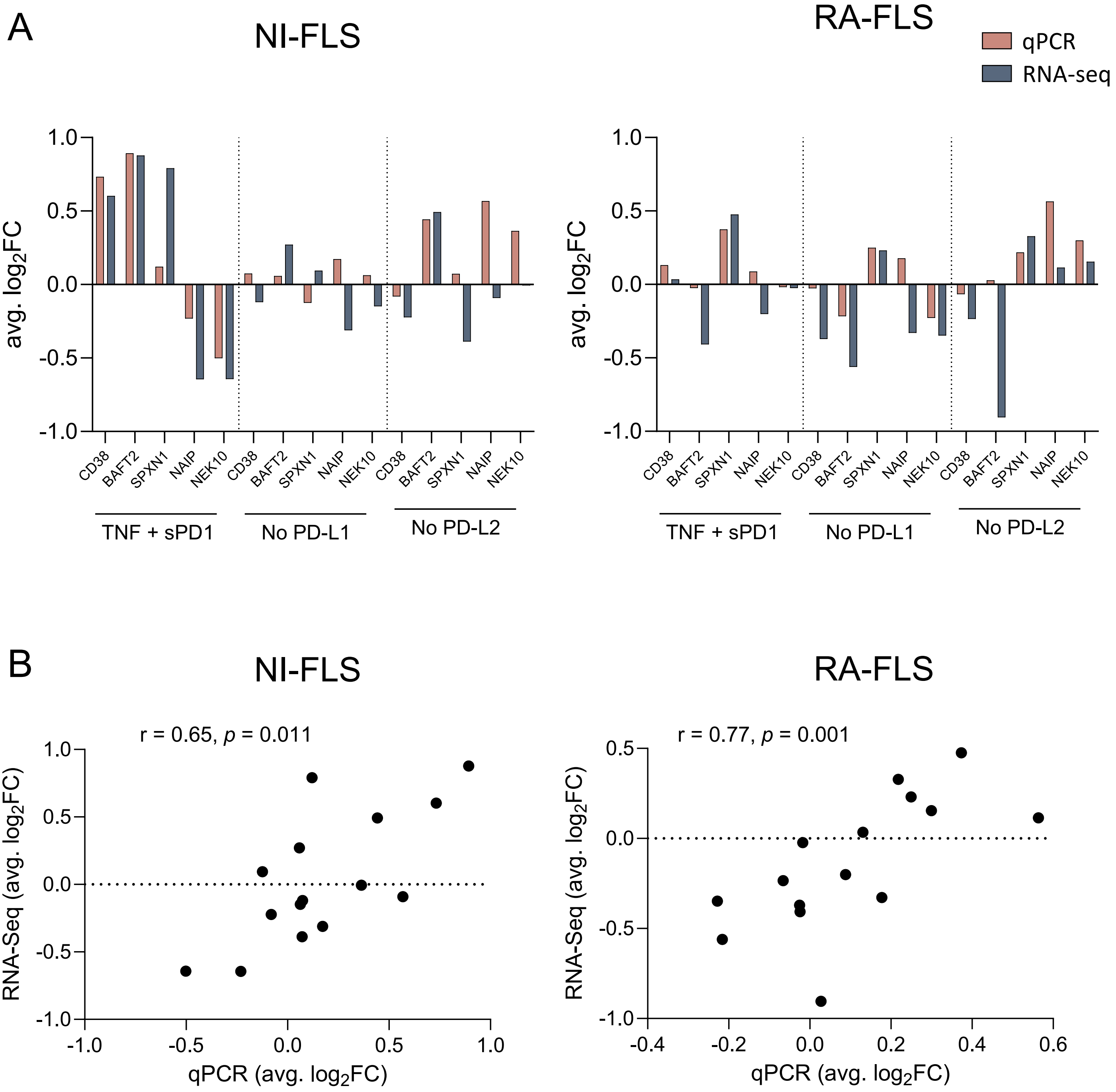

Supplementary Fig 9. qPCR validation of sPD1-responsive genes. (**A**) Relative mRNA expression of selected sPD1-responsive genes (CD38, BATF2, NEK10, NAIP, and SRXN1) measured by qPCR following TNF + sPD1 treatment in mock-transfected, PD-L1 knock-out, or PD-L2 knock-out NI-FLS (n=3) and RA-FLS (n=3). Gene expression is shown as fold-change (log_2FC) relative to TNF alone. (**B**) Correlation between log_2FC obtained from RNA-seq and qPCR for all selected genes and conditions in NI-FLS and RA-FLS, demonstrating concordance between the two methods.
